# Deciphering optical coupled resonant systems with physics-data co-driven deep neural networks

**DOI:** 10.1038/s41377-026-02389-0

**Published:** 2026-06-23

**Authors:** Song-Yi Liu, Hao-Tian Zhong, Xiao-Chong Yu, Bo-Lun Zhang, Liu-Yang Zhang, Yi Xu, Ning-Hua Zhu, Jin-hui Chen, Hua-Shun Wen, Shan-Guo Huang, Da-Quan Yang

**Affiliations:** 1 State Key Laboratory of Information Photonics and Optical Communications, Beijing University of Posts and Telecommunications, Beijing, China; 2 School of Information and Communication Engineering, Beijing University of Posts and Telecommunications, Beijing, China; 3 School of Mechanical Engineering, Xi’an Jiaotong University, Xi’an, Shaanxi China; 4 School of Physics and Astronomy, Key Laboratory of Multiscale Spin Physics (Beijing Normal University), Ministry of Education, and Beijing Key Laboratory of Advanced Metamaterial Structures and Functional Technologies, Beijing Normal University, Beijing, China; 5 Key Laboratory of Photonic Technology for Integrated Sensing and Communication, (Guangdong University of Technology), Ministry of Education of China, Guangzhou, China; 6 Institute of Intelligent Photonics, National Key Laboratory of Semiconductor Laser, Academy for Advanced Interdisciplinary Studies, Nankai University, Tianjin, China; 7 Institute of Electromagnetics and Acoustics, Xiamen University, Xiamen, China

**Keywords:** Physics, Optics and photonics

## Abstract

Coupled mode theory (CMT) is a universal method for studying resonant systems in various disciplines in science. Combined with traditional fitting methods, implicit physical parameters of the resonant systems can be revealed. However, this methodology fails in tackling the scenario of multi-solution for a given resonant system, resembling a fundamental challenge that has not been addressed yet. In this work, we propose and experimentally demonstrate a CMT physics and data co-driven deep neural network (CMT-NN) that can predict the implicit physical parameters of complex resonant systems in a rapid and precise way. More importantly, the challenge of multi-solution is mitigated by incorporating physical eigenvalues and response of the system in evaluating the physics consistency of the neural network. The applicability and generality of CMT-NN are demonstrated by simulations and experiments, where the CMT-NN can capture subtle spectral features and learn the coupling physical properties effectively. Compared with the traditional fitting method, the average computation time has been reduced by three orders of magnitude and the prediction performance is improved by more than two orders of magnitude. Displacement sensing experiments further validate the robustness of CMT-NN. It is anticipated that the CMT-NN can provide a paradigm shift in using the CMT for studying resonant systems and shed new light on the understanding, design and optimization of various coupled resonant systems.

## Abstract (web summary)

The physics-data co-driven deep neural network enables rapid and precise prediction of implicit physical parameters in complex coupled resonant systems, deciphering their underlying physical properties in a unified way.

## Graphical abstract

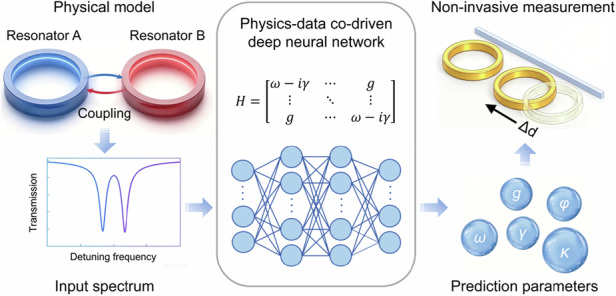

## Introduction

**Fig. 1:**
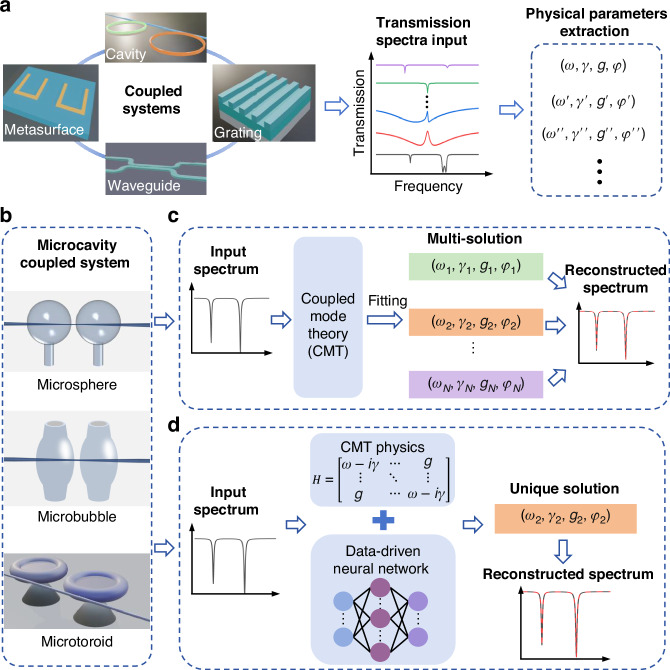
Diverse coupled systems analyzed by coupled mode theory (CMT). **a** Analyzing diverse coupled systems using the CMT, where *ω* (intrinsic resonant frequency), *γ* (loss), *g* (coupling strength) and *φ* (accumulated phase) are typical fitting physical parameters of the coupled systems. **b** Schematics of typical coupled resonant systems consisting of two microcavities, including but not limited to microspheres, microbubbles and microtoroids. **c** A schematic of the traditional fitting method for extracting the physical parameters using CMT, where multi-solution can result in the same transmission spectrum. **d** A schematic of CMT and data co-driven neural network for precise and fast prediction of physical parameters of the coupled system

Resonant coupling effects have become a fundamental physical concept in the study of exceptional point^1–3^, topology^4–6^, and bound states in the continuum^7–9^, significantly driving the development of high-performance sensing^10–13^, quantum computing^14,15^, and ultra-narrow lasers^16,17^. Coupled mode theory (CMT)^18–22^ has become a unified semi-analytical approach to understand the principles behind these intriguing coupling phenomena and manipulate the responses of the coupled systems. For example, CMT has been widely applied in the principle analysis of mechanical vibration systems^23,24^, optical systems^25–29^, circuit network systems^30,31^, quantum systems^32,33^, phononic systems^34^, etc. By fitting either the theoretically calculated or experimentally measured response of the coupled system utilizing the transmission formula derived from the CMT, the implicit physical parameters of the studied system can be computationally extracted. The state-of-the-art approach involves optimizing algorithms to fit the transmission spectra^35–37^, thus obtaining physical parameters such as resonance frequencies (*ω*), losses (*γ*), and coupling strengths (*g*) of diverse coupled resonant systems, as shown in Fig. 1a. However, this approach becomes invalid for a coupled resonant system with multi-solution of physical parameters for the same transmission spectrum, as shown in Fig. 1b, c, indicating a challenge in applying CMT for understanding the underlying physics of resonant systems. At the same time, the exponential growth of computational cost caused by searching in high-dimensional parameter space using optimization algorithms and indeterminate time-cost caused by human-supervised iterative optimization also impose great challenges in applying traditional fitting methods for retrieving the physical parameters. These challenges hinder the design of sophisticated coupled resonant systems and severely limit the application of CMT in different disciplines.

In recent years, the rapid development of deep learning and neuromorphic computing systems^38,39^ has revolutionarily transformed methodologies in the fields of physics^40–42^, chemistry^43^, biomedicine^44,45^, and information science^46,47^. It has become a new paradigm for solving inverse problems in science. Traditional deep neural networks (DNNs) can learn the end-to-end mapping relationships of unknown systems in a data-driven manner^48–50^. Though the performance of the data-driven DNN can be substantially improved by increasing the quantity and diversity of the training data, it still cannot tackle the problem of multi-solution. Combining deep learning algorithms with physical models has been proven to be an effective solution to release the burden of data^51–54^ and enable the processing of information^55,56^. However, the potential of employing physics-informed DNNs to solve the problem of multi-solution remains a compelling yet underexplored issue.

Herein, we propose and experimentally demonstrate a CMT and data co-driven neural network (CMT-NN) to mitigate the challenges of multi-solution and computational complexity. It is shown that the CMT-NN can efficiently and accurately predict the implicit physical parameters of typical coupled resonant systems using a transmission spectrum as an input, as shown in Fig. 1d. The incorporation of physical eigenvalues and transmission response of the resonant system to the neural network plays an important part in avoiding the solution multiplicity. In terms of batch spectral processing, both the theoretical calculation and experimental results show that the proposed method reduces computation time by approximately three orders of magnitude and improves the predicted accuracy by more than two orders of magnitude compared with traditional fitting methods. The robustness of CMT-NN is further validated by displacement sensing experiments. This work not only demonstrates a new paradigm of using CMT to analyze the underlying physics of coupled resonant systems but also provides flourishing prospects for high-precision sensing applications.

## Results

### Physical model of two-cavity coupled system

**Fig. 2:**
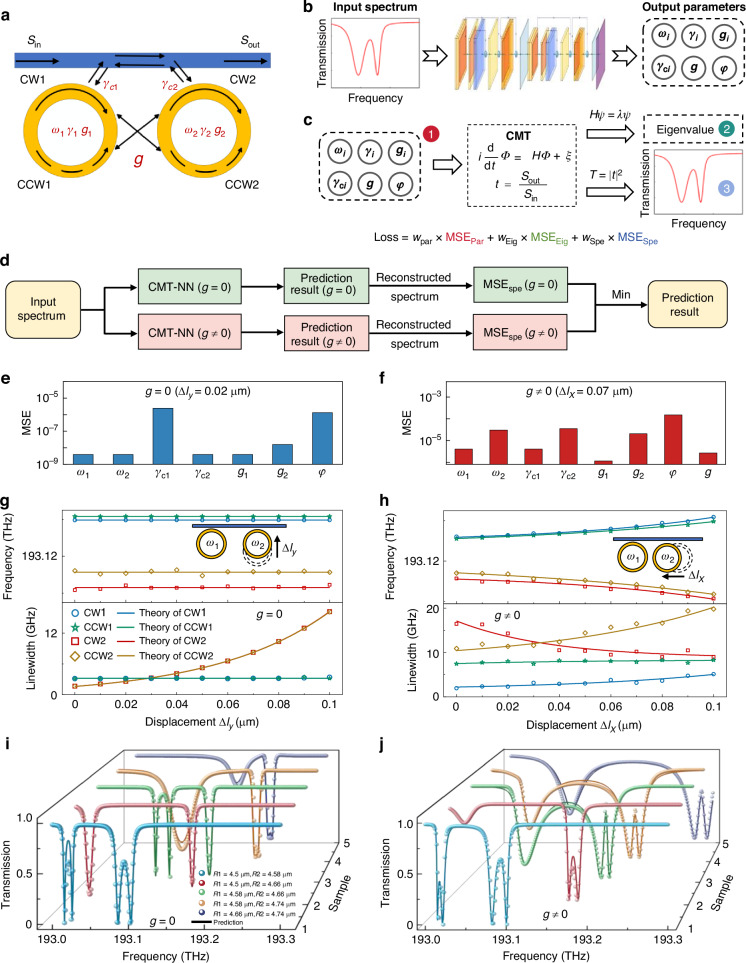
The framework and performance of the CMT and data co-driven neural network (CMT-NN). **a** A universal two-microring coupled resonant system served as a typical example. The black arrows represent the direction of energy flow. **b** The CMT-NN is used to predict the physical parameters of a coupled resonant system based on the input of a transmission spectrum. **c** The loss function consists of three parts: the physical parameters error, eigenvalues error and reconstructed transmission spectral error. The *w*_*i*_ is the weight for three parts. Par parameter, Eig eigenvalue, Spe spectrum. **d** The flowchart of CMT-NN for finding the physical parameters close to the ground truths. MSE mean squared error, Min minimum operation. **e**, **f** The errors of predicted parameters using CMT-NN when *g* = 0 and Δ*l*_*y*_ = 0.02 μm in (**g**) and *g* ≠ 0 and Δ*l*_*x*_ = 0.07 μm in (**h**), respectively. **g**, **h** The eigenvalues calculated using the predicted parameters of CMT-NN for two different coupling scenarios. Here, two microrings are without direct coupling and the displacement Δ*l*_*y*_ (i.e. parameter *γ*_c2_ is changed) is varied in (**g**). While the direct coupling is present and the displacement Δ*l*_*x*_ (i.e. parameters *g* and *φ* are both changed) is varied in (**h**), respectively. The hollow markers correspond to the eigenvalue results calculated using the predicted parameters of CMT-NN and the lines are the theoretical calculation results using the ground truth by CMT. The black arrows in insets represent directions of displacement Δ*l*_*y*_ and Δ*l*_*x*_ for the second microring. **i**, **j** The reconstructed spectra (solid lines) using the predicted physical parameters of CMT-NN and the transmission spectra simulated by FEM (spheres) for different microcavity sizes when direct coupling is absent (**i**) and present (**j**)

Without loss of generality, we consider a universal two-microcavity coupled system as an example, where two microrings are cascaded and coupled with a waveguide (see Fig. 2a). There are totally 4 resonant modes considered here, including a clockwise (CW) mode and a counterclockwise (CCW) mode of the first microcavity (Cav1) as well as a CW mode and a CCW mode of the second microcavity (Cav2). Energy exchange occurs between the two cavities through direct and indirect coupling. The CW (CCW) of the Cav1 is indirectly coupled with the CW (CCW) of the Cav2 through the waveguide, and the CW (CCW) of Cav1 directly couples with the CCW (CW) of Cav2. The effective Hamiltonian of this system is given as follows:

1 $$H=\left[\begin{array}{cccc}{\Delta }_{1}-i\frac{{\gamma }_{c1}+{\gamma }_{1}}{2} & {g}_{1} & 0 & g\\ {g}_{1} & {\Delta }_{1}-i\frac{{\gamma }_{c1}+{\gamma }_{1}}{2} & g & -i\sqrt{{\gamma }_{c1}{\gamma }_{c2}}{e}^{i\varphi }\\ -i\sqrt{{\gamma }_{c1}{\gamma }_{c2}}{e}^{i\varphi } & g & {\Delta }_{2}-i\frac{{\gamma }_{c2}+{\gamma }_{2}}{2} & {g}_{2}\\ g & 0 & {g}_{2} & {\Delta }_{2}-i\frac{{\gamma }_{c2}+{\gamma }_{2}}{2}\end{array}\right]$$

where Δ_1_ = *ω*_1_ − *ω*_0_ and Δ_2_ = *ω*_2_ − *ω*_0_ are the frequency detunings of two microcavities, *ω*_0_ is the reference frequency, *ω*_1_ and *ω*_2_ denote the intrinsic resonant frequencies of the two microcavities, respectively. *γ*_1_ (*γ*_2_) and *γ*_*c*1_ (*γ*_*c*2_) represent the intrinsic loss of the first (second) microcavity and the coupling efficiency between the first (second) microcavity and the waveguide, respectively. *g* indicates the direct evanescent coupling between two microcavities, while *g*_1_ (*g*_2_) represents the coupling coefficient between the CW and CCW of the first (second) microcavity. *φ* is the accumulated phase when the light wave travels between two microcavities. Based on the eigen equation $H\psi =\lambda \psi$(where *ψ* is the eigenstate of the Hamiltonian *H*), the eigenvalues of the corresponding coupled model can be evaluated. Four eigenfrequencies of the coupled system correspond to the real parts of the Hamiltonian eigenvalues, and the linewidths of the resonances are determined by the imaginary parts of the eigenvalues (details of the equations are provided in Supplementary Note 1).

### CMT and data co-driven neural network (CMT-NN)

Figure 2b illustrates the CMT-NN model that can predict the system’s physical parameters based on the transmission spectra (details of the network are provided in Supplementary Note 2). For the coupled system, the influence of multiple physical parameters on characteristics of transmission spectra is complex and diverse, manifesting itself as resonance mode degeneracy or splitting. This leads to an intricate nonlinear relationship between the implicit physical parameters of the coupled resonant system and the transmission spectrum, which can be learned by a DNN with an effective and diverse dataset. The CMT equations are used to theoretically calculate the transmission response of a two-microring coupled system under different physical parameters, establishing data pairs between transmission spectra and their corresponding physical parameters of the coupled system. The dataset is then constructed using the Latin hypercube sampling method^57^ (see Methods section for details). Unlike supervised neural networks, we incorporate physical constraints by employing a CMT-based physical model, which provides both eigenvalues and transmission response that are integrated into the loss function of DNN, as shown in Fig. 2c. It means that the entire pipeline of CMT-NN, from training to prediction, is grounded by physical principles. Specifically, the training dataset is entirely generated based on CMT, establishing a physically meaningful mapping among physical parameters, eigenvalues and transmission spectral responses, thereby addressing the large experimental data challenge for traditional DNNs. During the training of the DNN, the corresponding eigenvalues and transmission spectrum, calculated from the temporary outputs of the DNN using the CMT, are fed back to guide the backpropagation of the neural network. It means that the loss function of the DNN consists of two physically driven parts mediated by the CMT theory and a data-driven part (see Methods section for details), which can substantially avoid local optimum and achieve a unique prediction with high accuracy. The Fig. 2d shows the flowchart of CMT-NN model for finding the physical parameters close to the ground truths of an unknown transmission spectrum (see the Methods section for details). The combination of embedding the physical eigenvalues of the coupled system into the loss function and discriminating the minimum error of transmission spectrum can substantially avoid local optimum and achieve a unique solution with high accuracy.

### Simulation results for the CMT-NN

We first investigate the performance of the proposed CMT-NN trained by the dataset, which consists of calculated transmission spectra using the CMT and their corresponding parameters (see Methods section for simulation details). Figure 2e, f shows the normalized mean squared error (MSE) of the predicted physical parameters for two typical coupling cases, where the MSE is less than 10^–4^ at both the indirect coupling case (*g* = 0, Δ*l*_*y*_ = 0.02 μm) and direct evanescent coupling case (*g* ≠ 0, Δ*l*_*x*_ = 0.07 μm). The Δ*l*_*y*_ and Δ*l*_*x*_ represent the relative displacements of Cav2 under different scenarios, as indicated in the inset of Fig. 2g, h. Furthermore, Fig. 2g, h shows the eigenvalues calculated using the predicted parameters by CMT-NN for two different coupling scenarios that are without and with the direct coupling between two microcavities, respectively. When the direct coupling is absent (i.e. *g* = 0), the calculated results (hollow markers) match quite well with their ground truths as the distance Δ*l*_*y*_ between Cav2 and the waveguide (i.e. different *γ*_c2_) is changed (see Supplementary Note 3), indicating the eigenfrequencies and linewidths of the coupled resonant system excellently agree with the ones calculated using the ground truths of parameters, as shown in Fig. 2g. When the direct coupling is present, the CMT-NN is also capable of precisely predicting eigenfrequencies and linewidths of the coupled resonant system as the direct coupling coefficient *g* and the phase *φ* are both varied together with the increase of displacement Δ*l*_*x*_, as shown in Fig. 2h. To further demonstrate the performance of CMT-NN, we use the transmission spectra simulated by solving Maxwell equations using finite element method (FEM) as the input of CMT-NN to test the generality and robustness of the neural network. As can be seen in Fig. 2i, j, the reconstructed spectra (solid lines) using the predicted physical parameters of CMT-NN and Supplementary Eq. (S7) agree quite well with the FEM results (spheres) for both coupling cases with different microcavity sizes, showcasing the excellent accuracy of predicted physical parameters for the transmission spectra not belonging to the test dataset. Because the CMT is a very universal theory for handling coupled resonant systems, a dataset generated using the CMT can be used to train the neural network to learn the fundamental physical mechanisms, enabling it to predict the physical parameters using the inputs from the FEM simulation data. These results indicate that the CMT-NN model can accurately capture the spectral features, predict the system’s physical parameters with high accuracy and generality, and achieve precise spectral reconstruction for resonant systems with different coupling scenarios.

### Performance comparisons between the CMT-NN and other DNNs

**Fig. 3:**
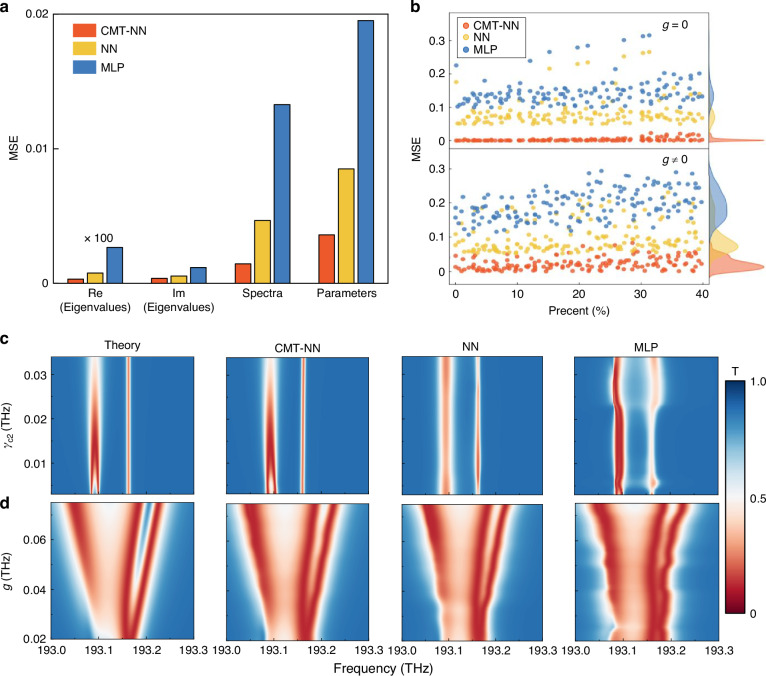
Comparison of performances between the CMT-NN and two typical deep neural networks. **a** Comparison of three cases in MSE of eigenvalues (imaginary and real part), reconstructed transmission spectra using the predicted physical parameters and physical parameters of CMT-NN, CMT-NN when its physics-driven parts of eigenvalue and transmission spectra are not included (NN) and multilayer perceptron (MLP) in the validation set. The errors for the eigenvalues’ real part have been magnified by 100 times for visibility. Re real, Im imaginary. **b** The MSE of predicted physical parameters when one of the parameters is outside the range of the training and validation dataset (the deviation is 0–40%) for two different coupling scenarios. Each data point represents the average error calculated from 25 spectra. The plots on the right axis represent the statistical distributions of errors. For better visualization, the errors of NN (yellow circle) and MLP (blue circle) are shifted up by 0.05 and 0.1, respectively. **c**, **d** Reconstructed transmission spectra using the predicted physical parameters of CMT-NN (the second column), NN (the third column) and MLP (the rightmost panels) methods with the input of transmission spectra (the leftmost panels). Here, *γ*_c2_ and *g* are varying in (**c**) and (**d**), respectively, where *ω*_1_ = 193.0944 THz, *ω*_2_ = 193.1614 THz, *γ*_c1_ = 0.0063 THz, *g*_1_ = 0.0081 THz, *g*_2_ = 0.002 THz, *g* = 0 THz, *φ* = 3.078 rad, *γ*_1_ = 0.3 GHz, *γ*_2_ = 0.2 GHz are used in (**c**). And *ω*_1_ = 193.09 THz, *ω*_2_ = 193.164 THz, *γ*_c1_ = 0.04 THz, *γ*_c2_ = 0.0215 THz, *g*_1_ = 0.007 THz, *g*_2_ = 0.003 THz, *φ* = 1.4 rad, *γ*_1_ = 0.3 GHz, *γ*_2_ = 0.2 GHz are used in (**d**). The color bar is also provided

In order to provide quantitative comparisons with solely data-driven neural networks, we also investigate the performance of the neural network used in CMT-NN when its physics-driven parts of eigenvalue and transmission spectra are not included (NN)^58,59^ on the same dataset. The performance of commonly used the multilayer perceptron^60,61^ (MLP) model is also compared (see Methods section and Supplementary Note 2 for more details). Based on the predicted results from the validation set shown in Fig. 3a, the average errors of eigenvalues, transmission spectra and physical parameters for the CMT-NN are substantially smaller than the other two cases. Compared with the NN/MLP, the predicted errors of eigenvalues, transmission spectra, and physical parameters are decreased by 32.76%/69.30%, 68.91%/89.06%, and 57.71%/81.59%, respectively. These substantial improvements in the accuracy of predicted parameters and intermediate physical constraints prevent the CMT-NN from falling to local optimum (see Supplementary Note 4 for more comparison details). To further validate the generalization ability of CMT-NN, we test the cases of physical parameters that are outside the range of the training and validation dataset, as shown in Fig. 3b. Compared with the NN and MLP models, the predicted results of CMT-NN (red points) exhibit the smallest MSE and the narrowest statistical distributions of errors for predicted physical parameters at two different coupling scenarios, when the deviation percentage of the parameters is 0–40% (see Methods section for details). The MSEs of CMT-NN (2.838 × 10^−3^ with *g* = 0, 2.279 × 10^−2^ with *g* ≠ 0) are reduced by about one order of magnitude and the variance of error distribution (2.235 × 10^−5^ with *g* = 0, 3.209 × 10^−4^ with *g* ≠ 0) is reduced up to two orders of magnitude compared with the other two cases. It is worth noting that the CMT-NN demonstrates a good agreement between its predicted MSEs for both the validation set as well as the one beyond the training and validation datasets, as shown in Fig. 3a, b, respectively. This indicates extrapolation stability without overfitting and parameter saturation (the training and validation loss dynamics of CMT-NN are provided in Supplementary Fig. S3).

These results indicate that the CMT-NN not only inherits the excellent generalization ability of data-driven neural networks but also effectively possesses the precision endowed by the physics-driven neural network, showcasing the superiority of co-driven neural network by data and CMT physics. Furthermore, we used these models to predict the corresponding physical parameters for continually varying transmission spectra, where the predicted parameters are then used to reconstruct the transmission spectra by CMT. As shown in the second columns of Fig. 3c, d, the reconstructed spectra using physical parameters predicted by CMT-NN match quite well with the input spectra (the first columns of Fig. 3c, d), indicating excellent predicted accuracy of CMT-NN. In stark contrast, the transmission spectra predicted by the other two models exhibit large deviations (see Supplementary Note 4 for more comparison details). These results substantially validate that the physics and data co-driven CMT-NN exhibits unique advantages in accuracy and generality.

### Performance comparisons between the CMT-NN and the traditional fitting method

**Fig. 4:**
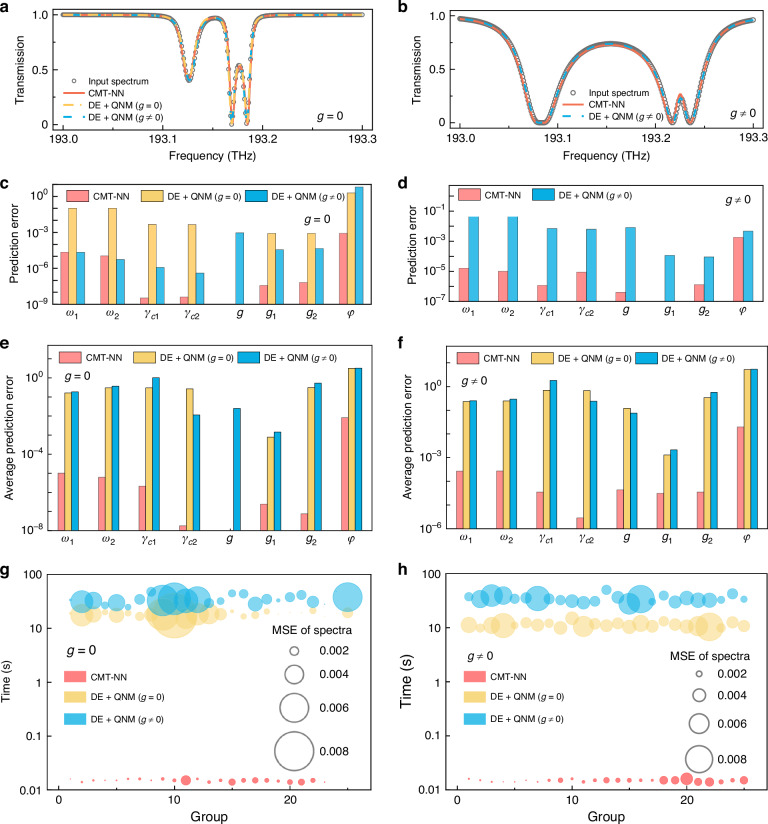
Comparison of performance between the CMT-NN and the traditional fitting method (differential evolution quasi-Newton method, DE + QNM). **a**, **b** Two typical examples where the reconstructed transmission spectra using the CMT-NN, the traditional fitting method for the cases with (*g* ≠ 0) and without (*g* = 0) direct coupling. The circles, red lines, blue lines and yellow lines correspond to the input transmission spectra, reconstructed transmission spectra using the predicted physical parameters of CMT-NN, fitting transmission spectra by DE + QNM (*g* ≠ 0) and DE + QNM (*g* = 0), respectively. **c**, **d** The corresponding MSE of the predicted physical parameters by CMT-NN, DE + QNM (*g* ≠ 0) and DE + QNM (*g* = 0) for the cases shown in (**a**) and (**b**). **e**, **f** The average predicted errors of physical parameters in 250 realizations for CMT-NN, DE + QNM (*g* ≠ 0) and DE + QNM (*g* = 0) in the case of (**e**) *g* = 0 and (**f**) *g* ≠ 0. **g**, **h** The average computation time and mean reconstructed spectral errors in 25 groups for CMT-NN, DE + QNM (*g* ≠ 0) and DE + QNM (*g* = 0) under the scenarios of *g* = 0 (**g**) and *g* ≠ 0 (**h**). The size of circle shows the mean reconstructed spectral error

Falling into local optimum and being time-consuming are typical challenges for the traditional fitting method. We further compare the performance in predicted accuracy and computation time between the CMT-NN model and the traditional fitting method of differential evolution quasi-Newton method^62^ (DE + QNM) (details of the fitting method are provided in Supplementary Note 5), where random transmission spectra with different coupling conditions from the validation set are used. For a transmission spectrum of an unknown coupled resonant system, the traditional fitting method needs to find the optimal solutions of both *g* = 0 and *g* ≠ 0, which naturally results in multi-solution. Each solution corresponds to a completely different coupled system, which in turn poses a great challenge in understanding the coupled system. Two typical examples are given in Fig. 4a, b, the reconstructed spectra of different methods using their predicted physical parameters show good consistency with the input transmission spectra. However, there are significant differences in the errors of physical parameters, as shown in Fig. 4c, d. For the input transmission spectrum shown in Fig. 4a, the normalized mean parameter errors of CMT-NN, DE + QNM (*g* = 0) and DE + QNM (*g* ≠ 0) are 10^−4^, 7.398 × 10^−1^, 2.691 × 10^−1^, respectively, as shown in Fig. 4c. It means that a resonant system with direct coupling (i.e. *g* ≠ 0) is deduced using the traditional fitting method, where the CMT-NN can precisely predict that there is no direct coupling between two cavities (i.e. *g* = 0). For the input transmission spectrum shown in Fig. 4b, the mean parameter errors of CMT-NN and DE + QNM (*g* ≠ 0) are 2 × 10^−4^ and 1.45 × 10^−2^, respectively, as shown in Fig. 4d. It should be noted that the case of DE + QNM (*g* = 0) cannot fit the input spectrum when the direct coupling is present (data not shown). As can be seen from Fig. 4c, d, the accuracy of predicted parameters using CMT-NN is two to three orders of magnitude improved compared with the traditional fitting method. These results confirm the existence of multi-solution in extracting the physical parameters using traditional fitting method, while the physics and data co-driven CMT-NN can effectively mitigate this issue (see Supplementary Table S2 for more details about fitting and predicted parameters).

To further consolidate the advantages of CMT-NN compared with the traditional fitting method, we randomly select 250 transmission spectra from the validation set and divide them into 25 groups for both coupling cases. It should be noted that we show the errors of physical parameters, reconstructed spectra and computation time excluding human-supervised iterative optimization for a fair comparison between the CMT-NN and traditional fitting method. As shown in Fig. 4e, f, the CMT-NN’s mean MSEs of predicted parameters are 1 × 10^−3^ and 2.5 × 10^−3^, respectively, where the predicted accuracy is improved by more than two orders of magnitude compared with the traditional fitting method in 250 transmission spectra under both coupling cases (i.e. *g* = 0 and *g* ≠ 0). Furthermore, compared with the average computation time of DE + QNM (14.8323 s, *g* = 0) and DE + QNM (34.6094 s, *g* ≠ 0), the average computation time of CMT-NN is only 0.0148 s, which is reduced by three orders of magnitude. And the mean reconstructed spectral error of CMT-NN is 8.05 × 10^−4^, where the accuracy is improved by 74.34% and 77.67% for the two coupling scenarios, respectively, as shown in Fig. 4g, h. These results validate that CMT-NN possesses faster computation time and higher accuracy for all the 25 groups, demonstrating the unparalleled advantages of CMT-NN.

### Experimental validation and displacement sensing applications

**Fig. 5:**
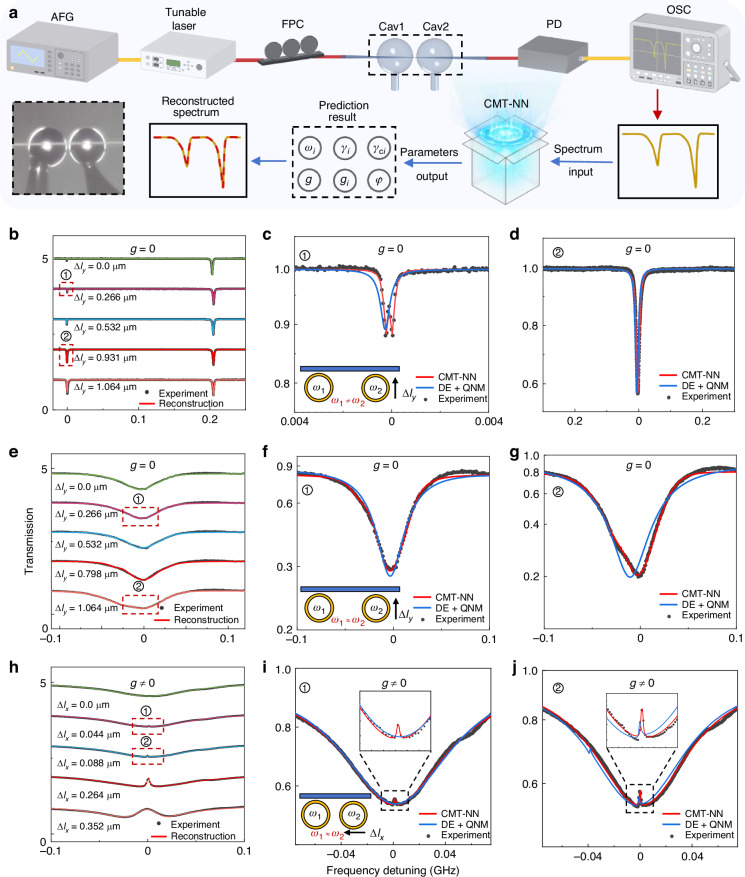
Experimental validation of the two-microsphere coupled resonant system. **a** Schematic of experimental setup. AFG arbitrary function generator, FPC fiber polarization controller, PD photodetector, OSC oscilloscope. **b**–**d** Reconstructed transmission spectra using the predicted physical parameters of CMT-NN with different *γ*_c2_ when the resonant frequencies of two microspheres are not equal (i.e. *ω*_1_ ≠ *ω*_2_) and the direct coupling between two microspheres is zero (i.e. *g* = 0). Two typical examples are magnified in (**c**) and (**d**). **e**–**g** Reconstructed transmission spectra using predicted physical parameters of CMT-NN with different γ_c2_ when the resonant frequencies of two microspheres are similar (i.e. *ω*_1_ ≈ *ω*_2_) and the direct coupling between two microspheres is zero (i.e. *g* = 0). Two typical examples are magnified in (**f**) and (**g**). **h**–**j** Reconstructed transmission spectra using predicted physical parameters of CMT-NN with different direct coupling *g* when the resonant frequencies of two microspheres are similar (i.e. *ω*_1_ ≈ *ω*_2_) and the coupling between Cav2 and waveguide is zero (i.e. *γ*_c2_ = 0). Two typical examples are magnified in (**i**) and (**j**). The insets show the magnification of the resonant peak. The points indicate experimentally measured transmission spectra, the red lines are the reconstructed transmission spectra using the parameters predicted by CMT-NN, and the blue lines are the fitted transmission spectra

Experimental validation for predicting the physical parameters of a coupled system using the measured transmission spectrum is performed in the coupled microspheres system, as shown in Fig. 5a. The experimental setup is also shown in Fig. 5a (see the Methods section for details), where the CMT-NN is trained by theoretical calculation data using the CMT. Figure 5b–d shows the reconstructed transmission spectra (red lines) using the predicted parameters of CMT-NN for different resonant frequencies of two microspheres, when the distance Δ*l*_*y*_ (the range is 0–1.064 μm) between Cav2 and the tapered fiber (i.e. different *γ*_c2_) is changed. Here, the direct coupling between two microcavities is zero (i.e. *ω*_1_ ≠ *ω*_2_ and *g* = 0) in Fig. 5b–d. It can be seen from Fig. 5b that the agreement between the experimentally measured (points) and reconstructed transmission spectra (red lines) using the predicted parameters of CMT-NN is excellent under different Δ*l*_*y*_. As can be seen from the two typical examples in Fig. 5c, d, the reconstructed transmission spectra for the coupling scenarios that are with mode splitting (Fig. 5c) and mode degeneracy (Fig. 5d) match quite well with the experimentally measured spectra. It can also be seen that the traditional fitting method cannot resolve the mode splitting, indicating the superiority of using the CMT-NN for extracting the physical parameters of coupled resonant systems.

To further demonstrate the superior performance of CMT-NN, the case of similar resonant frequencies (i.e. *ω*_1_ ≈ *ω*_2_) is considered as well. Figure 5e–j shows the reconstructed transmission spectra using the predicted parameters of CMT-NN. As shown in Fig. 5e–g, the reconstructed transmission spectra using the predicted parameters of CMT-NN agree much better with the experimental ones than the traditional fitting method when the direct coupling is absent (*g* = 0). Furthermore, the reconstructed spectra using the predicted parameters of CMT-NN also show good consistency with the experimental results when the distance Δ*l*_*x*_ (the range is 0–0.352 μm) between two microspheres (i.e. different *g*) is changed and the direct coupling between Cav2 and waveguide is zero (i.e. *γ*_c2_ = 0), as shown in Fig. 5h. The CMT-NN can well reconstruct the experimental spectra, especially for some sharp spectral features (red lines in Fig. 5i, j). In stark contrast, the reconstructed transmission spectra by the traditional fitting method cannot capture the key features of resonances. It should be emphasized that the CMT-NN for extracting physical parameters in experiment is trained by the theoretical calculation dataset incorporating Gaussian noise (see Methods), which improves the prediction accuracy, especially for the sharp features of transmission spectra. These results further pinpoint the generality and robustness of CMT-NN. Furthermore, the experimental system can maintain long-term stability, since thermal drift is negligible (see Supplementary Note 6). These results consolidate that the CMT-NN is capable of precisely predicting physical parameters for coupled resonant systems and reconstructing intricate features of the transmission spectra.

**Fig. 6:**
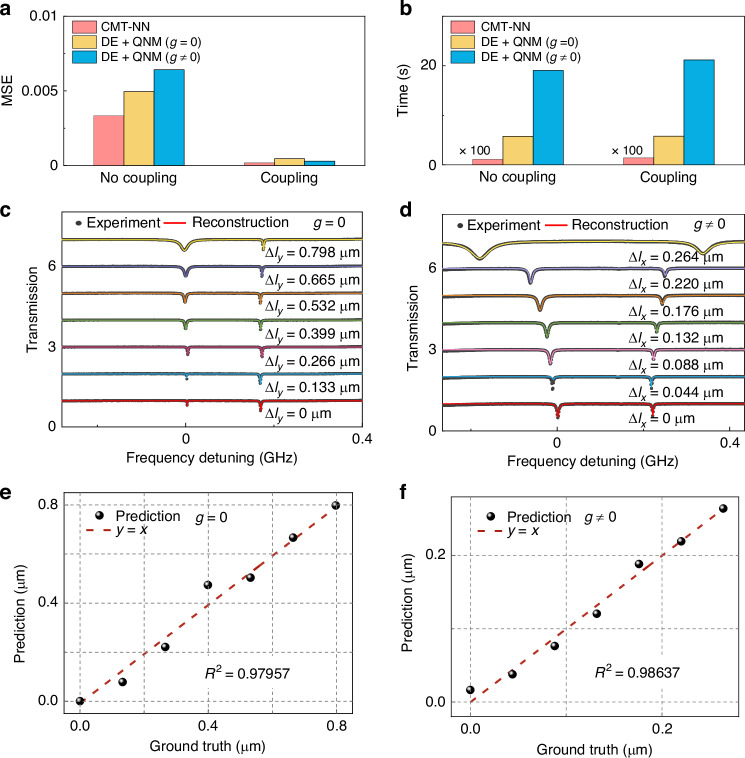
Experimental results and displacement sensing applications. **a**, **b** The mean reconstructed spectral error and the corresponding average computation time of the CMT-NN, DE + QNM (*g* ≠ 0) and DE + QNM (*g* = 0) for the cases without and with direct coupling in the test set, respectively. The computation time for CMT-NN have been magnified by 100 times for readability. **c**, **d** The displacement-dependent transmission spectra acquired in experiment (points) and the corresponding reconstructed spectra using the parameters predicted by CMT-NN (lines) for two different displacement sensing scenarios (i.e. displacements of Δ*l*_*y*_ (**c**) and Δ*l*_*x*_ (**d**) of Cav2 shown in the inset of Fig. 2g, h). **e**, **f** Dependence of predicted displacements Δ*l*_*y*_ (**e**) and Δ*l*_*x*_ (**f**) on their ground truths

We further quantitatively compare the performance of CMT-NN with the traditional fitting methods in experiment, as shown in Fig. 6a. For the experimental transmission spectra of the test set, the mean reconstructed spectral error of CMT-NN is 1.99 × 10^−3^ which is comparable to yet marginally larger than the simulation ones in Fig. 4g, h, showcasing the effectiveness of CMT-NN in experiment. The enlargement of the mean reconstructed spectral error is also because of noise in experiment. It should be noted that the ground truths of the physical parameters for the coupled systems cannot be obtained in the experimental scenario, so the errors of the reconstructed transmission spectra are used instead. Furthermore, compared with the cases of DE + QNM (5.7803 s, *g* = 0) and DE + QNM (20.1319 s, *g* ≠ 0), the average computation time of CMT-NN is only 0.0125 s which is reduced up to three orders of magnitude. These results validate that the traditional fitting method becomes invalid for the coupled system with multi-solution, while the CMT-NN model can accurately predict physical parameters close to the ground truths for the coupled resonant system.

Due to capability of CMT-NN to obtain the unique solution close to the ground truth, we validate its application in displacement sensing (see the Methods section for experiment details). The input transmission spectra and the reconstructed spectra for the first displacement scenario where the Cav2 is gradually moved close to the tapered fiber (i.e. different displacement Δ*l*_*y*_) are shown in Fig. 6c. While the corresponding results of the second displacement scenario where the Cav2 is gradually moved close to the Cav1 (i.e. different displacement Δ*l*_*x*_) are shown in Fig. 6d. All the reconstructed spectra using the predicted physical parameters of CMT-NN show good agreement with the input spectra, although slight deviations may appear in the positions of certain resonant modes. The deviation can be reduced by using the training dataset which combines the experimental data with the calculated one. In addition, Fig. 6e, f shows that the predicted displacements Δ*l*_*y*_ and Δ*l*_*x*_ also agree well with ground truths, where the MSEs of predicted displacement are 1.6 × 10^−3^ and 1.0453 × 10^−4^, respectively. The corresponding coefficients of determination R² of displacement sensing for the two cases are 0.97957 and 0.98637, respectively, indicating excellent performance of displacement sensing. To further validate the consistency of displacement sensing results under different microcavity geometries and fabrication tolerances, we perform additional validations. The experimental results demonstrate that the CMT-NN maintains high performance (with R^2^ values up to 0.9964) under varying geometries and fabrication batches (see Supplementary Note 7 for more details), confirming its excellent robustness.

## Discussion

We introduce and demonstrate experimentally the concept of CMT-NN for physical parameter prediction in complex coupled resonant systems, where the challenges of multi-solution and computational complexity are effectively mitigated. By combining both physical eigenvalues and response of the system mediated by the CMT with the data-driven neural network, key implicit physical parameters of coupled resonant systems can be effectively and precisely extracted. The CMT-NN with high predicted accuracy can output unique physical parameters according to one input spectrum while the traditional fitting method will inevitably yield multi-solution, providing a new paradigm in studying coupled systems. Compared with the traditional fitting method, the average computation time is reduced by three orders of magnitude while the predicted accuracy is improved by more than two orders of magnitude. The applicability of CMT-NN in both the FEM simulation data and experimental data consolidates the robustness of CMT-NN in handling data from different sources. Ultimately, the CMT-NN model can be generalized to various sensing applications using coupled resonant systems. The CMT-NN is particularly suitable for large-scale data tasks with its rapid inference capability and high prediction accuracy, such as real-time dynamic monitoring and high-throughput device characterization. For a typical application, CMT-NN enables non-invasive quantitative characterization of on-chip optical resonant systems, offering a potential alternative to conventional approaches (such as scanning electron microscope) for characterization of device morphology in the future. In addition, by expanding the data space with multi-parameter sensing spectra or incorporating sensing matrix^11^ as the physical constraint, the framework can simultaneously disentangle and quantify multiple physical parameters from a single complex spectrum, thereby enabling multi-parameter sensing.

The performance of CMT-NN can be further improved by more advanced neural network models based on self-supervised learning^63^, pre-trained denoising techniques^64^, and a broader range of physical features (such as eigenvectors and amplitudes)^18^. Considering non‑Gaussian systematic errors in specific experimental environments, transfer learning^51^ can be employed to fine‑tune the model with a small amount of target‑scene experimental data, allowing rapid adaptation to the specific error distribution without full retraining. Furthermore, the CMT-NN framework is also applicable to multi-cavity coupled systems, particularly those with topological structures. The structural symmetry of the system can be used to avoid significant increases in neural network depth and data requirements. For general multi-cavity systems, training data can be efficiently generated using CMT, while strategies such as few-shot learning^65^ and data augmentation^66^ further reduce data dependency. At the same time, the introduction of architectural optimization methods, such as residual neural networks^67^, comprehensive normalization^58^, and dropout regularization^68^, could effectively mitigate issues of gradient disappearance or parameter saturation, ensuring both the applicability and training stability of the model in complex multi-cavity systems. Moreover, the generality of CMT-NN allows it to be extended to other resonant systems, where it can be used to explore new phenomena in complex coupled systems with nonlinear effects^69–71^ and provide new possibilities for applications. This is because the CMT-NN is generally applicable to physical systems whose theory or empirical response can be embedded into the loss function. Even simplified or imperfect physical models can guide the network to convergence and preserve prediction accuracy^53^. This generality establishes the CMT-NN as an alternative approach for parameter inversion and demonstrates promising potential for extension to a broader range of physical systems.

## Methods

### Dataset generation

In general, the dynamic evolution of coupled resonant systems can be modeled by the CMT and the training datasets in simulation and experiment results are both generated by the CMT. For the training dataset in simulation, the ranges for parameters *ω*_1_, *ω*_2_, *γ*_c1_, *γ*_c2_, *g*_1_ and *g*_2_ are 193.015–193.19, 193.12–193.25, 0–0.05, 0.001–0.05, 0–0.02 and 0–0.01 THz, respectively. The direct coupling coefficient *g* ranges from 0 to 0.1 THz, and the phase *φ* ranges from 0 to 2π rad. The intrinsic losses *γ*_1_ and *γ*_2_ are fixed at 0.3 and 0.2 GHz, respectively. 31.5k data pairs are generated, with 18.1k data pairs corresponding to *g* = 0 and the other 13.4k data pairs corresponding to *g* ≠ 0. For the training dataset in experiment, the ranges for parameters Δ_1_, Δ_2_, *γ*_1_, *γ*_2_, *γ*_c1_, *γ*_c2_, *g*_1_ and *g*_2_ are −0.02–0.1, −0.02–0.22, 0–0.07, 0–0.028, 0–0.09, 0–0.05, 0–0.015, 0–0.015 GHz, respectively. The direct coupling coefficient *g* ranges from 0 to 0.015 GHz, and the phase *φ* ranges from 0 to 2π rad. 52k data pairs are generated, with 19.7k data pairs corresponding to *g* = 0 and the other 32.3k data pairs corresponding to *g* ≠ 0. The transmission spectra of simulation and experiment utilize 301 and 2501 sampling points in frequency, respectively, ensuring complete preservation of resonance features. Additional Gaussian noise is introduced to the generated dataset to simulate noise introduced by the experimental environment and equipment. All the datasets are divided into a training set (70%), a validation set (30%) and a test set (the simulation dataset consists of simulation transmission spectra and the experiment dataset consists of 38 measured transmission spectra).

### Loss function

In the CMT-NN, we use the following loss function:

2 $$\begin{array}{l}L{\mathrm{oss}}_{{\mathrm{total}}}=\frac{1}{N}\mathop{\sum}\limits_{i=1}^{N}{w}_{p,i}({p}_{i}-{\hat{p}}_{i}{)}^{2}+{w}_{{\rm{e}}}\frac{1}{M}\mathop{\sum}\limits_{j=1}^{M}[({\mathrm{Re}}({\lambda}_{j}-{\hat{\lambda}}_{j}))^{2}\\\qquad\qquad\,\,\,\,+\,({\mathrm{Im}}({\lambda}_{j}-{\hat{\lambda}}_{j}))^{2}{]}+{w}_{s}\frac{1}{P}\mathop{\sum }\limits_{{\rm{k}}=1}^{P}{({s}_{k}-{\hat{s}}_{k})}^{2}\end{array}$$

where *w* represents weights of different loss terms, *p*_*i*_ represents the predicted value of the physical parameter, $\hat{{p}_{i}}$ represents the ground truth of the physical parameter, *N* = 8 is the number of parameters. And *w*_*e*_ represents the weight of eigenvalue loss, λ_*j*_ represents the predicted value of the complex eigenvalue, $\hat{{\lambda }_{j}}$ represents the ground truth corresponding to that predicted value, and *M* = 4 is the number of eigenvalues. While *w*_*s*_ represents the weight of spectral loss, *s*_*k*_ represents the reconstructed transmission spectrum, $\hat{{s}_{k}}$ represents the input transmission spectrum, and *P* is the number of sampling points per transmission spectrum. All the weights in the loss function are adaptively optimized during the training process. Specifically, we utilize a separate Adam optimizer dedicated solely to the learnable loss weights, dynamically adjusting their values while enforcing a strict lower bound (i.e. 0.2) for all weights. This prevents any individual loss term from being excessively weakened or neglected during training, thereby effectively avoiding the model’s collapse to a trivial solution (the weights of different losses dynamics of CMT-NN are provided in Supplementary Fig. S5).

In the NN and MLP model, the loss function is used:

3 $$L{\rm{oss}}=\frac{1}{N}\mathop{\sum }\limits_{i=1}^{N}({p}_{i}-{\hat{p}}_{i}{)}^{2}$$

### Training of CMT-NN

The CMT-NN, NN and the MLP are implemented in PyTorch 2.5.1 platform with Python 3.12.7, deployed on a workstation equipped with an AMD Ryzen 9 7950X CPU and NVIDIA RTX4070TI SUPER GPU. We adopt the Adam optimizer with cosine annealing learning rate scheduling, where the initial learning rate is set to 1 × 10^−4^ and gradually decays to 1 × 10^−6^ through the training cycles to optimize the weights. The CMT-NN, NN and MLP are trained for 300 epochs. The activation function of CMT-NN and NN is GELU, and MLP’s activation function is ReLU. Considering the significant differences in data distributions and spectral features between direct coupling (*g* ≠ 0) and indirect coupling (*g* = 0), the training process of CMT-NN is divided into two parts: *g* = 0 and *g* ≠ 0. To further enhance the network capacity and training performance, the regularization strategies and early stopping can be employed to reduce the risk of overfitting^68,72^.

### Finding the physical parameters close to the ground truth using the CMT-NN

For a transmission spectrum of an unknown coupled resonant system, we use the dual-path approach to output the physical parameters close to the ground truths. It means that the transmission spectrum under study is input to two well-trained CMT-NNs with and without direct coupling, as shown in Fig. 2d. The final output of the physical parameters is determined through the comparison of the spectral errors between their reconstructed spectra and the input spectrum, where the physical parameters with smaller error will be output.

### Full wave simulation

The simulation data are obtained by solving the Maxwell equations using the FEM. By adjusting the size of different microrings (i.e. different *ω*_*i*_), the distance between microrings and waveguide (i.e. different *γ*_ci_), and the distance between two microrings (i.e. different *g* and *φ*), the corresponding transmission spectra are obtained. The waveguide widths of microring and bus waveguide are both 0.35 μm. The outer radius of the microcavity ranges from 4.5 to 4.74 μm, and the step size is 0.08 μm for tailoring the resonant frequency. When direct coupling is absent, the distance between the second cavity and the waveguide ranges from 0.24 to 0.14 μm, and the step size is 0.01 μm. The distance between the two microcavities ranges from 3.5 to 4.5 μm, and the step size is 0.1 μm. When direct coupling is present, the distance between the two microcavities ranges from 0.15 to 0.25 μm, and the step size is 0.01 μm.

### Test data beyond the training and validation datasets

The percentage beyond the range of the training dataset shown in Fig. 3b is defined as the relative ratio of the physical parameter exceeding the parameter range defined in the previous Methods section of *Dataset generation*. For example, 10% indicates one of the parameters is 1.1 times the maximal value of the physical parameter’s range. The other parameters are randomly assigned values within the range of 0 to 1. The case where the physical parameters *ω*_1_ and *ω*_2_ exceed the dataset range is not considered here.

### Experimental details

The experimental system is shown in Fig. 5a. A tunable laser (Toptica DLC 1500) with a wavelength of 1550 nm is used to excite the resonant modes of the microspheres via the tapered fiber. The laser wavelength is precisely scanned using a 50 Hz triangular wave generated by an arbitrary function generator (AFG, Keysight 33600A). A fiber polarization controller (FPC) is employed to adjust the polarization state of the incident light to ensure optimal coupling efficiency. The transmission spectra are recorded in real-time with a low-noise photodetector (PD, KY-BPRM-200M-I-FA) and monitored on an oscilloscope (OSC, Rigol DHO4804). A three-dimensional nano-positioning platform is used to accurately control the distance Δ*l*_*y*_ between the microspheres and the tapered fiber (i.e., parameter *γ*_c2_ is changed), as well as the distance Δ*l*_*x*_ between the two microspheres (i.e., direct coupling coefficient *g* and the accumulated phase *φ* are both changed). Cav1 is attached to the tapered fiber (i.e., in an over-coupled state) to ensure the stability of the system. In experiment, we actively reduce the incident optical power to effectively suppress thermally induced asymmetric spectral distortion. Additionally, we acquire the background spectrum as a baseline to calibrate the transmission spectra prior to coupling the microcavities with the fiber taper, thereby effectively eliminating the influence of background fluctuations.

## Supplementary information

Supplementary Information for Deciphering optical coupled resonant systems with physics-data co-driven deep neural networks

## Data Availability

The data that support the findings of this study are available from the corresponding authors upon request.

## Code availability

The Python codes used in this paper are available at https://github.com/ezriaz/CMT-NN.
