# Supplementary material for: Deciphering optical coupled resonant systems with physics-data co-driven deep neural networks
Source: Light Sci Appl. 2026 Jun 23;15:279. doi: 10.1038/s41377-026-02389-0 (PMC13291268; doi:10.1038/s41377-026-02389-0)
Supplement: Supplementary file 1 — Supplementary Information for Deciphering optical coupled resonant systems with physics-data co-driven deep neural networks [file 41377_2026_2389_MOESM1_ESM.docx]

**Supplementary Information for**

**Deciphering optical coupled resonant systems with physics-data co-driven deep neural networks**

Song-Yi Liu^1,2,8^, Hao-Tian Zhong^3,8^, Xiao-Chong Yu^4,8^, Bo-Lun Zhang^1,2^, Liu-Yang Zhang^3^, Yi Xu^5,^*, Ning-Hua Zhu^6^, Jin-hui Chen^7^, Hua-Shun Wen^6,^*, Shan-Guo Huang^1^, Da-Quan Yang^1,2,^*

^1^State Key Laboratory of Information Photonics and Optical Communications, Beijing University of Posts and Telecommunications, Beijing, 100876, China.

^2^School of Information and Communication Engineering, Beijing University of Posts and Telecommunications, Beijing, 100876, China.

^3^School of Mechanical Engineering, Xi’an Jiaotong University, Xi’an, Shaanxi, 710049, China.

^4^School of Physics and Astronomy, Key Laboratory of Multiscale Spin Physics (Beijing Normal University), Ministry of Education, and Beijing Key Laboratory of Advanced Metamaterial Structures and Functional Technologies, Beijing Normal University, Beijing 100875, China.

^5^Key Laboratory of Photonic Technology for Integrated Sensing and Communication (Guangdong University of Technology), Ministry of Education of China, Guangzhou, 510006, China.

^6^Institute of Intelligent Photonics, National Key Laboratory of Semiconductor Laser, Academy for Advanced Interdisciplinary Studies, Nankai University, Tianjin, 300071, China.

^7^Institute of Electromagnetics and Acoustics, Xiamen University, Xiamen 361005, China.

^8^These authors contributed equally to this work.

*Correspondence: yixu@gdut.edu.cn (Y.X.), whs@nankai.edu.cn (H.-S.W.), ydq@bupt.edu.cn (D.-Q.Y.)

**Supplementary Note 1 — Hamiltonian of the two-microcavity coupled resonant system**

We build a theoretical model based on the coupled mode theory^1^ (CMT) to study the scattering and coupling behavior of the coupled microcavity system. The physical model of a two-microcavity direct coupled resonant system is shown in Fig. 2a in the main text. The transient equations of the coupled system are as follows:

$\frac{da_{1CW}}{dt}=-i\omega_{1}a_{1CW}-\frac{\gamma_{c1}+\gamma_{1}}{2}a_{1CW}-ig_{1}a_{1CCW}-iga_{2CCW}-\sqrt{\gamma_{c1}}S_{in}$ (S1)

 (S2)

 (S3)

$\frac{da_{2CCW}}{dt}=-i\omega_{2}a_{2CCW}-\frac{\gamma_{c2}+\gamma_{2}}{2}a_{2CCW}-ig_{2}a_{2CW}-iga_{1CW}$ (S4)

where *a_jCW_* (*a_jCCW_*) is the annihilation operator of the *j*-th (*j*=1,2) microcavity’s CW mode (CCW mode), with intrinsic resonant frequency $\omega_{j}$. $\gamma_{j}$, $\gamma_{cj}$ represent the intrinsic loss of *j*-th microcavity and the coupling coefficient between *j*-th microcavity and the bus waveguide respectively. *g_j_* is the couple strength between CW and CCW in the *j*-th microcavity. $\varphi$ is the accumulated phase when the light wave travels between two microcavities, and *g* is the direct evanescent coupling between two microcavities.

According to Equations (S1-S4), the equation of motion for the coupled system can be expressed as:

$i\frac{d}{dt}\Phi=H\Phi+\zeta$ (S5)

where $\Phi=\left( a_{1CW},a_{1CCW},a_{2CW},a_{2CCW} \right)^{T}$denotes the annihilation operator of the four traveling wave modes, $\zeta$ is the amplitude of energy coupled into the modes. *H* is the effective Hamiltonian of the coupled resonant system, the expression is given in Equation (1) of the main text.

In order to further analyze the transmission characteristics of the coupled system, we calculate the equation of transmission spectrum based on Equations (S1)-(S4):

 (S6)

The transmission spectrum of the two-microcavity coupled resonant system is expressed as follows:

 (S7)

where

 (S8)

Equation (S7) can be used to fit the spectra and calculate reconstructed transmission spectra by predicted parameters. Notably, benefiting from the ultra-high quality factor (~10^8^) of the fabricated microcavities and the precisely controlled undercoupling condition, we directly observe the internal coupling between the CW and CCW modes within the microcavity. This coupling manifests as a clear mode splitting phenomenon, as shown in Fig. 5c of the main text, which is a feature that can be accurately captured and described by the theoretical framework established above. These observations confirm that the coupling between CW and CCW modes is indispensable for the accurate description of our system.

**Supplementary Note 2 — The framework of CMT and data co-driven neural network (CMT-NN) and two typical deep neural networks**

In this section, we introduce the CMT and data co-driven neural network (CMT-NN) while its physics-driven parts of eigenvalue and transmission spectra are deleted (NN) and multilayer perceptron (MLP) models’ network architectures, as shown in Figures S1 and S2. The input and output layers of the three networks (CMT-NN, NN and MLP) are designed to process spectral data and predict the physical parameters, respectively. To better accommodate the one-dimensional spectral signals, we revise the original convolutional architecture by replacing its two-dimensional convolutional operations with one-dimensional convolutional operations, ensuring compatibility with the physical characteristics of the spectral data. NN model uses the multiple visual attention network (VAN) encoder blocks to handle the parameter extraction task^2^, as shown in Figure S1. In each encoder block, the input data is first mapped to a higher-dimensional space through an embedding process, during which the data's sampling rate is reduced, and batch normalization is applied to the data. Then, a large kernel attention mechanism is used to enhance the data processing capability. This mechanism primarily employs dilated convolution and deep convolution. Next, the enhanced high-dimensional spectral data is fed into the feed-forward module, which is implemented through deep convolution layers to enhance its feature learning ability. The core role of residual connection is to solve the problem of gradient disappearance/explosion in deep neural networks and alleviate network degradation, so as to improve model training efficiency and performance. After L iterations of attention extraction and feature learning, the data flows into the next encoder block, and the process of L iterations is repeated. Finally, key optical features are extracted through the global average pooling (GAP) operation, and the predicted physical parameters are output. This choice is motivated by the fact that a flattening layer would retain all spectral details, which could potentially lead the CMT-NN to overfit on the training set. The use of GAP effectively mitigates this risk. It stabilizes training and enhances model generalization for multi-parameter regression tasks by aggregating spectral features^3^, thereby reducing parameter complexity and decoupling the interdependence among output neurons. The L value in each encoder block is variable with the goal of enhancing the network's learning depth and expressive capability. It should be noted that the CMT-NN designs independent sub-networks for each physical parameter to address a limitation of the traditional fully connected layers. This strategy ensures that all parameters can converge to their optimal values, preventing cross-parameter interference in the learning process.

Figure S2 shows that the MLP model consists of a 9-layer multilayer perceptron with the activation function being ReLU^4^. The following number of neurons in each layer is used: 301, 400, 500, 500, 500, 400, 200, 100, 8. And inputs of the NN and MLP are 301 amplitudes of the transmission spectrum, and the outputs consist of 8 physical parameters. The loss function of NN and MLP is evaluated using errors of the physical parameters (see Methods section for detail in main text).


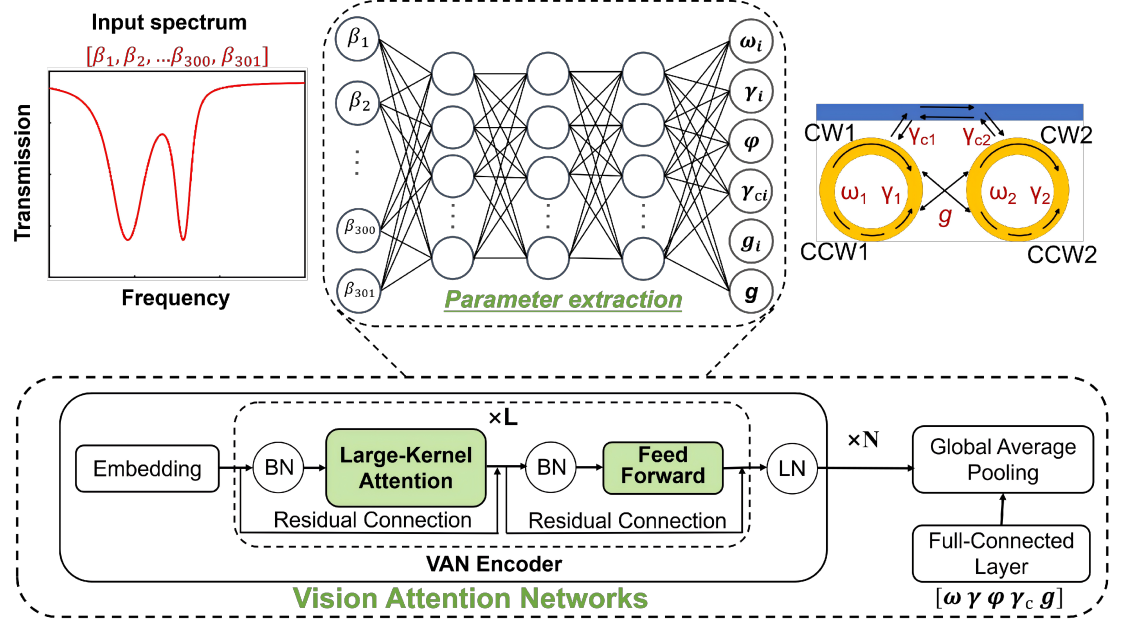


**Figure S1. The framework of NN model.** BN: Batch Normalization. LN: Layer Normalization.


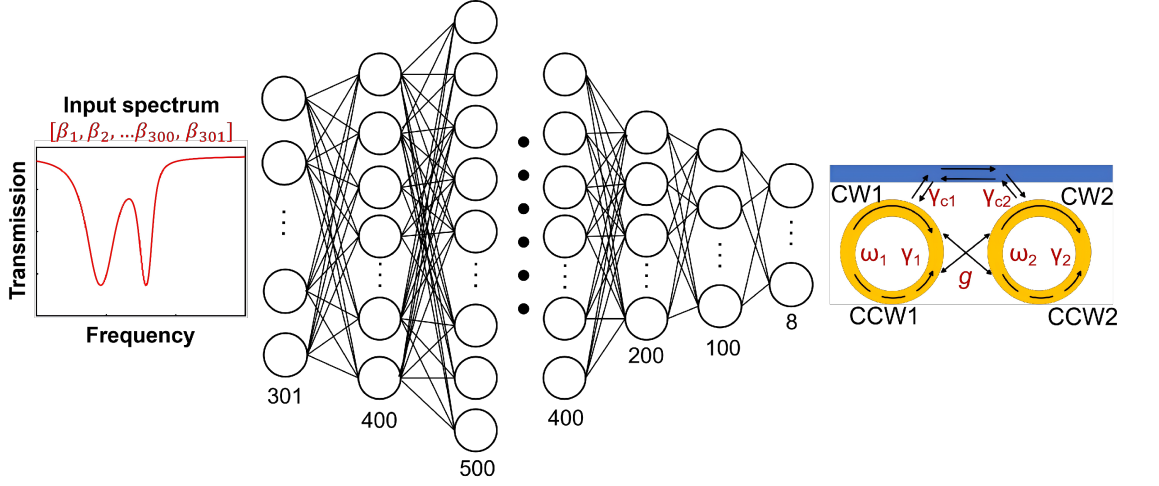


**Figure S2. The framework of MLP model.** The MLP model consists of 9 layers with the following number of neurons in each layer is used: 301, 400, 500, 500, 500, 400, 200, 100, 8.

Figure S3 shows the training and validation loss dynamics of CMT-NN with epochs. Both curves exhibit a rapid exponential decay during the initial 50 epochs, and the loss converges smoothly to a steady state after approximately 150 epochs. The final training loss and the validation loss of CMT-NN are 0.00052 and 0.00059 for *g* = 0, while they are 0.001 and 0.013 for *g* ≠ 0. The validation loss remains consistent with the training loss throughout the process, indicating good generalization capability and no apparent overfitting is observed. While for both the NN and MLP as shown in Figure S4, the training loss converges smoothly to a steady state, the validation loss begins to gradually increase after approximately 30 epochs, indicating overfitting occurs.


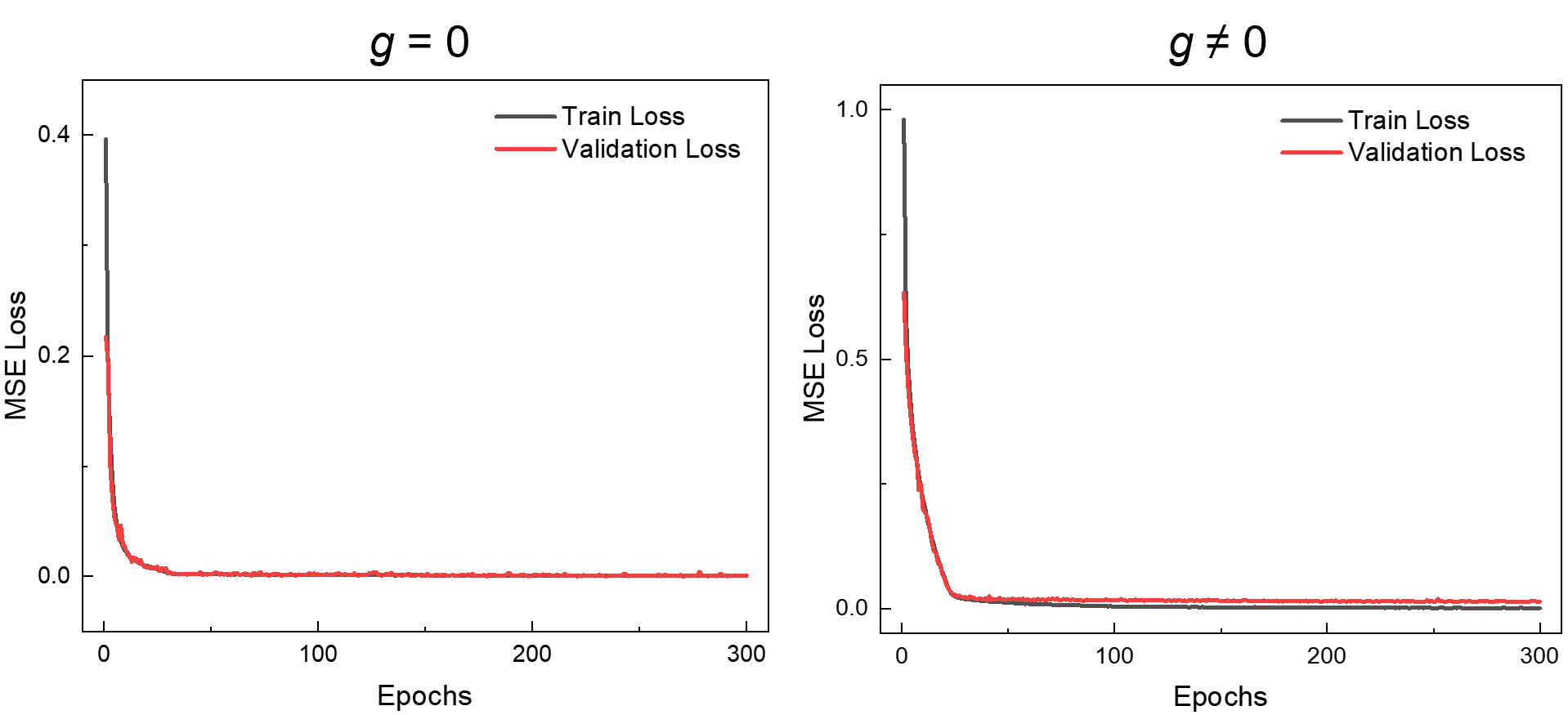


**Figure S3. The training and validation loss dynamics of CMT-NN when direct coupling is absent (*g* = 0) and present (*g ≠* 0).**


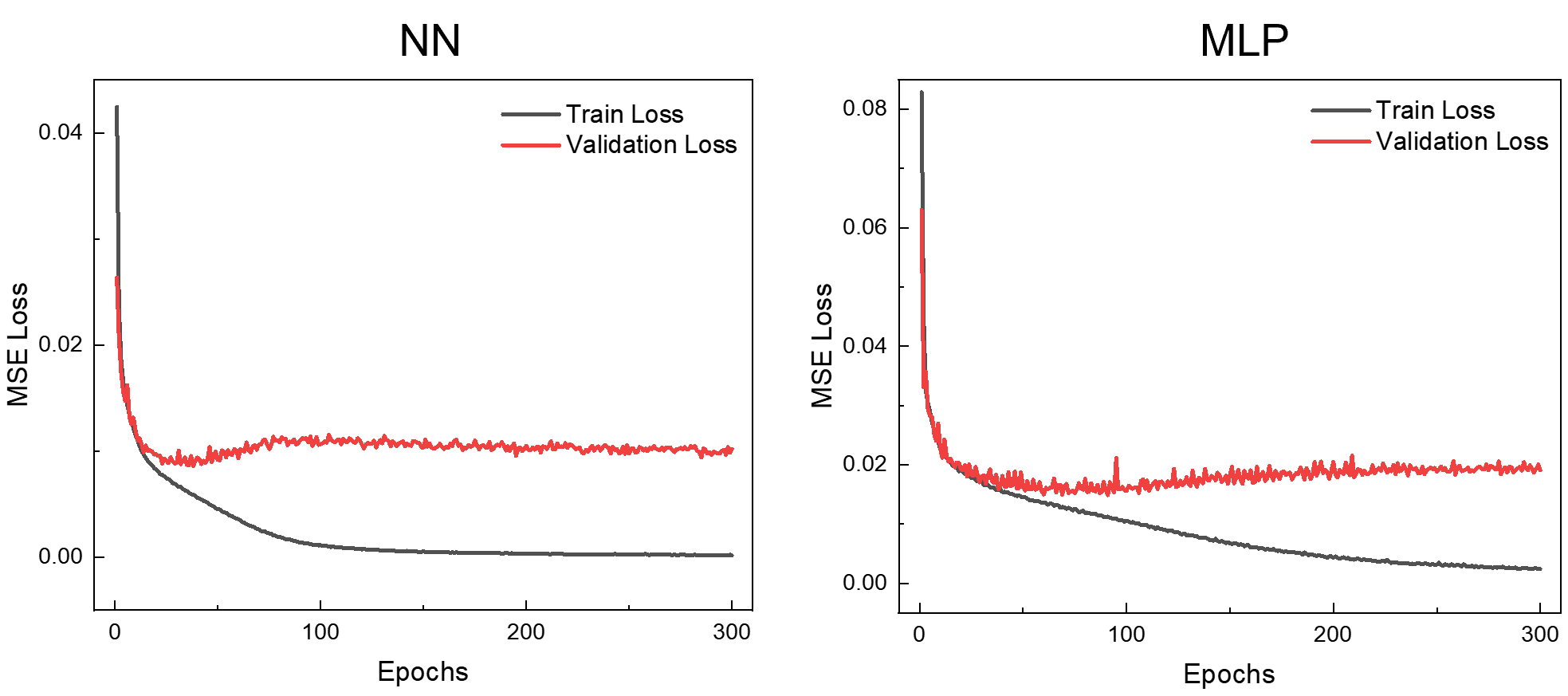


**Figure S4. The training and validation loss dynamics of NN and MLP.**

Figure S5 shows the dynamics of weights for different loss terms. The weight of spectral loss decreases first to its threshold (i.e. 0.2) and remains stable throughout the subsequent training process. While the weight corresponding to the eigenvalue loss is the last to reach stability. All weights are convergent after training in two coupling scenarios. These results indicate that the proposed adaptive weighting strategy provides effective physical guidance continuously during training, thereby successfully preventing the model from converging to a trivial solution.

**Figure S5. The dynamics of different loss weights of CMT-NN when direct coupling is absent (g = 0) and present (g ≠ 0).**

**Supplementary Note 3 — Predicted physical parameters’ errors of CMT-NN models for the simulated spectra of two-microcavity coupled resonant system**

In order to demonstrate the superiority of the CMT-NN’s performance, we use the simulated transmission spectra with varying displacements Δ*l_y_* and Δ*l_x_* for testing, and the errors of predicted physical parameters are shown in Figure S6. When the direct coupling is absent (i.e. *g* = 0), the predicted parameters match quite well with their ground truths as the distance between the second microcavity (Cav2) and the waveguide (i.e. different γ_c2_) is changed. The mean squared error (MSE) of predicted physical parameters is 3.2983×10^-5^. The right panel of Figure S6 shows CMT-NN is also capable of precisely predicting physical parameters of the coupled resonant system even when the direct coupling coefficient *g* and the phase *φ* are both changed together with the increase of displacement Δ*l_x_*, and MSE of predicted parameters is 9.894×10^-5^*.*


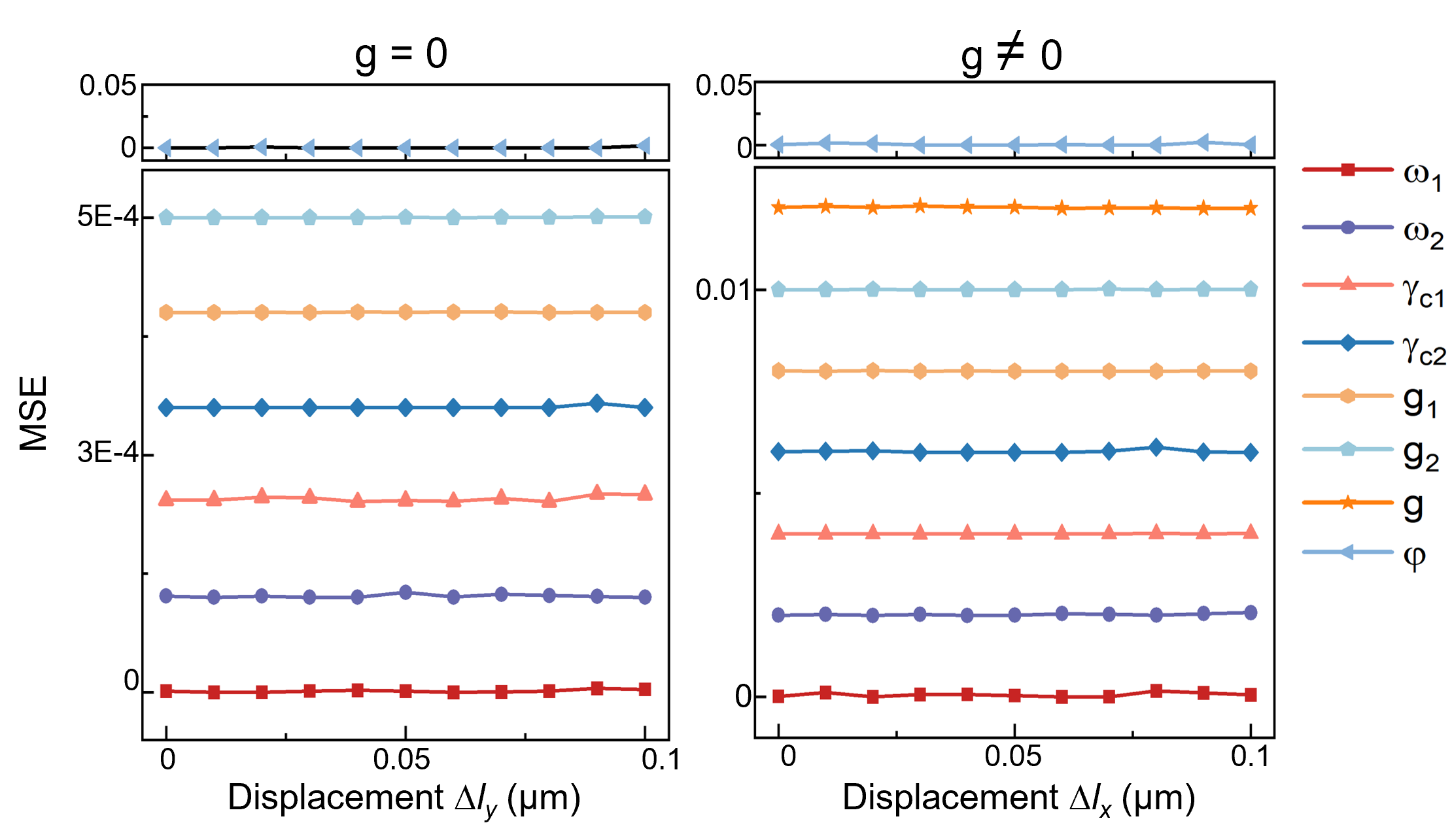


**Figure S6. Predicted parameter errors of CMT-NN model for simulated spectra in Figs. 2g and 2h.** For better visualization, the errors of such parameters (with the exception of *ω*_1_ and *φ*) are shifted up by integer multiples of 0.0001 (*g* = 0) and 0.002 (*g* ≠ 0), respectively. MSE: Mean squared error.

**Supplementary Note 4 — Performance comparison among the CMT-NN, NN and MLP models**

In this section, we investigate the performance of CMT-NN, NN and MLP by comparing the ability to reconstruct spectrum used predicted physical parameters, as shown in Figures S7 and S8. As shown in the second column of Figure S7, the reconstructed spectra using predicted physical parameters of CMT-NN match quite well with the input simulated transmission spectra by solving Maxwell equations using finite element method (the first column of Figure S7). The CMT-NN also achieves better predicted performance than the other two models. It can also be seen that the reconstructed spectra by NN and MLP models have different degrees of distortion compared to the input spectra. Further, we use these models to predict the corresponding physical parameters (i.e. parameters *γ*_c2_ and *φ*) for continually varying transmission spectra. As shown in Figures S8a and S8b, the reconstructed spectra of CMT-NN excellently agree with the input theoretical calculated spectra (the first columns of Figure S8a and S8b), indicating the accuracy of parameter prediction. In stark contrast, the transmission spectra predicted by the other two models exhibit significant deviation. These results indicate that the CMT-NN model can accurately capture the spectral features, and achieves precise spectral reconstruction for diverse coupled resonant systems.


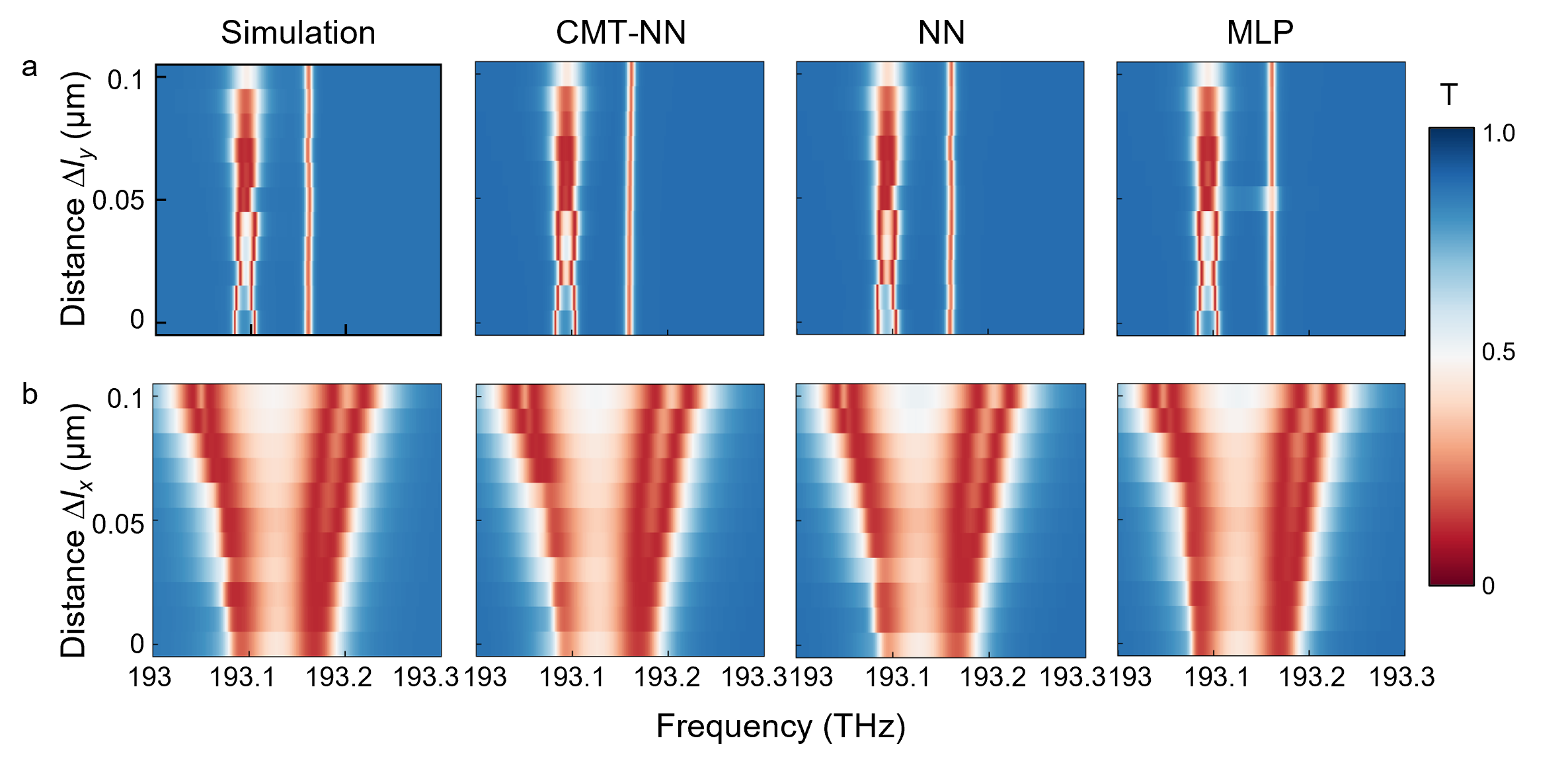


**Figure S7. Performance comparison among the CMT-NN, NN and MLP models for the reconstruction of simulated transmission spectra. a,** The reconstructed spectra of three models when the direct coupling strength *g* between two microrings is zero (i.e. *g* = 0) and the distance Δ*l_y_* between Cav2 and the waveguide (i.e. different *γ*_c2_) is changed. **b,** The reconstructed spectra of three models when the direct coupling strength *g* between two microrings is present (i.e. *g* ≠ 0) and the distance Δ*l_x_* between two cavities is changed (i.e. different *g* and *φ*). The simulated transmission spectra by finite element method and reconstructed transmission spectra of CMT-NN, NN and MLP models are provided, respectively. The colorbar of the transmission is provided.


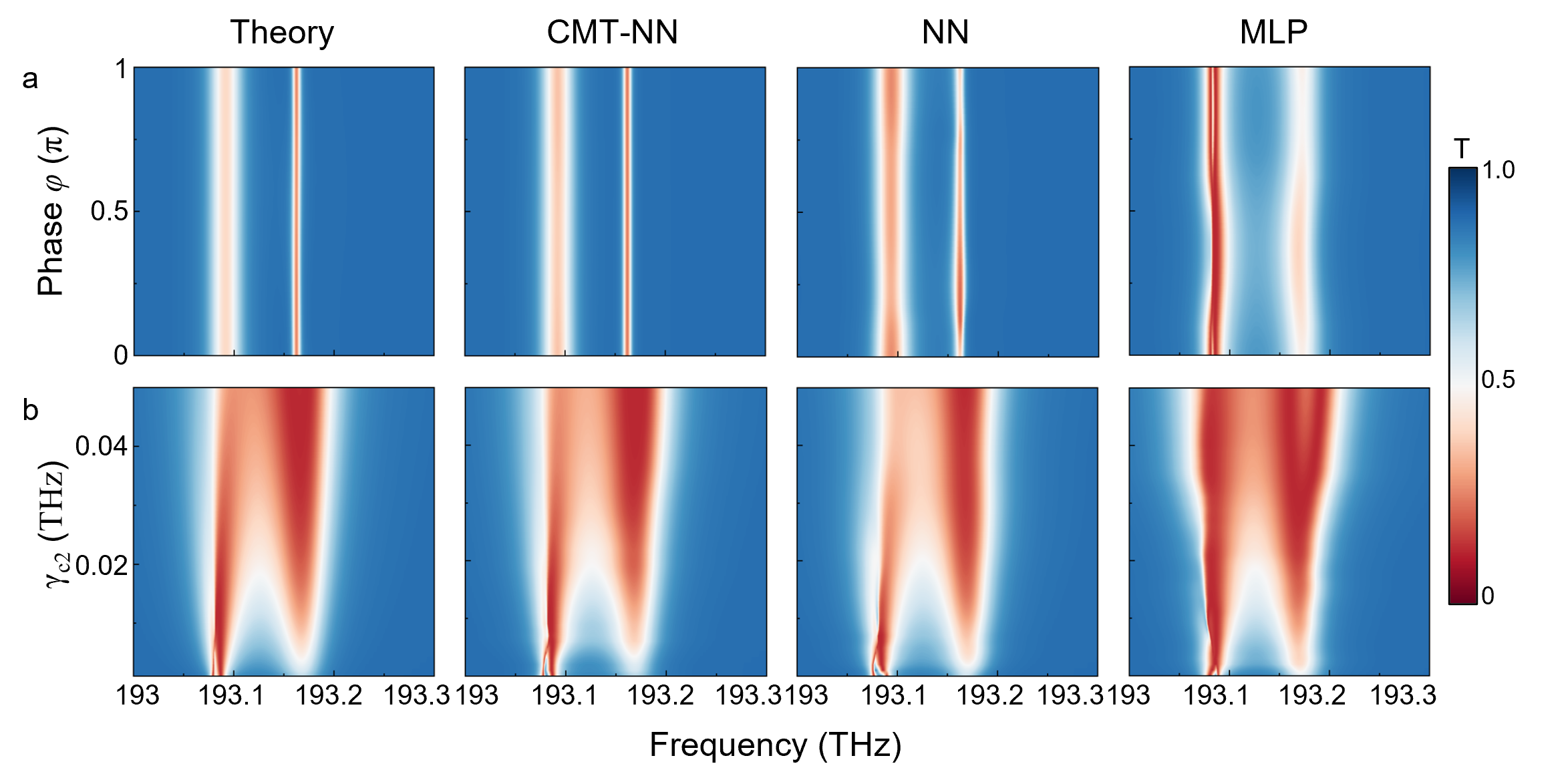


**Figure S8. Comparison among the CMT-NN, NN and MLP models for reconstruction of theoretical calculated spectra.** The calculated transmission spectra by CMT and reconstructed transmission spectra using predicted parameters of CMT-NN, NN and MLP models are provided, respectively. **a,** *ω*_1_ = 193.0944 THz, *ω*_2_ = 193.1614 THz, *γ*_c1_ = 0.0063 THz, *γ*_c2_ = 0.0185 THz, *g*_1_ = 0.0081 THz, *g*_2_ = 0.002 THz, *γ*_1_ = 0.3 GHz, *γ*_2_ = 0.2 GHz. **b,** *ω*_1_ = 193.09 THz, *ω*_2_ = 193.164 THz, *γ*_c1_ = 0.04 THz, *g*_1_ = 0.007 THz, *g*_2_ = 0.003 THz, *g* = 0.0475 THz, *φ* = 1.4 rad, *γ*_1_ = 0.3 GHz, *γ*_2_ = 0.2 GHz. The color bar of the transmission is provided.

To demonstrate the performance of CMT-NN, we investigate the capability of three models in determining the coupling conditions of the resonant system on the same validation set. As can be seen in Figure S9, the reconstructed spectra of CMT-NN and NN using their predicted physical parameters show consistency with the input transmission spectra. However, there are significant differences in the predicted parameter *g*, as shown in Table S1. For the input transmission spectrum shown in Figure S9a, the predicted direct evanescent coupling’s absolute errors of CMT-NN, NN and MLP are 0, 2.93464×10^-5^ and 0.0013, respectively. It means that a resonant system with direct coupling (i.e. *g* ≠ 0) is deduced using the other deep neural networks, while the CMT-NN can precisely predict that there is no direct coupling between two cavities (i.e. *g* = 0). Furthermore, for the resonant system with direct coupling (as shown in Figure S9b), the data-driven network MLP misjudges the coupling conditions, the predicted direct evanescent coupling’s absolute errors of CMT-NN, NN and MLP are 2.49671×10^-4^, 0.00694 and 0.01101, respectively. Beyond this representative case, statistical analysis across the entire validation set further confirms the superiority of CMT-NN. The mean absolute errors for the physical parameter *g* of CMT-NN, NN and MLP are 0/2.10×10^-3^, 1.65×10^-5^/3.07×10^-3^ and 3.49×10^-5^/6.09×10^-3^ in the case of without/with direct coupling, respectively. These results confirm that the CMT-NN can effectively improve the accuracy of characterizing the system's coupling conditions.


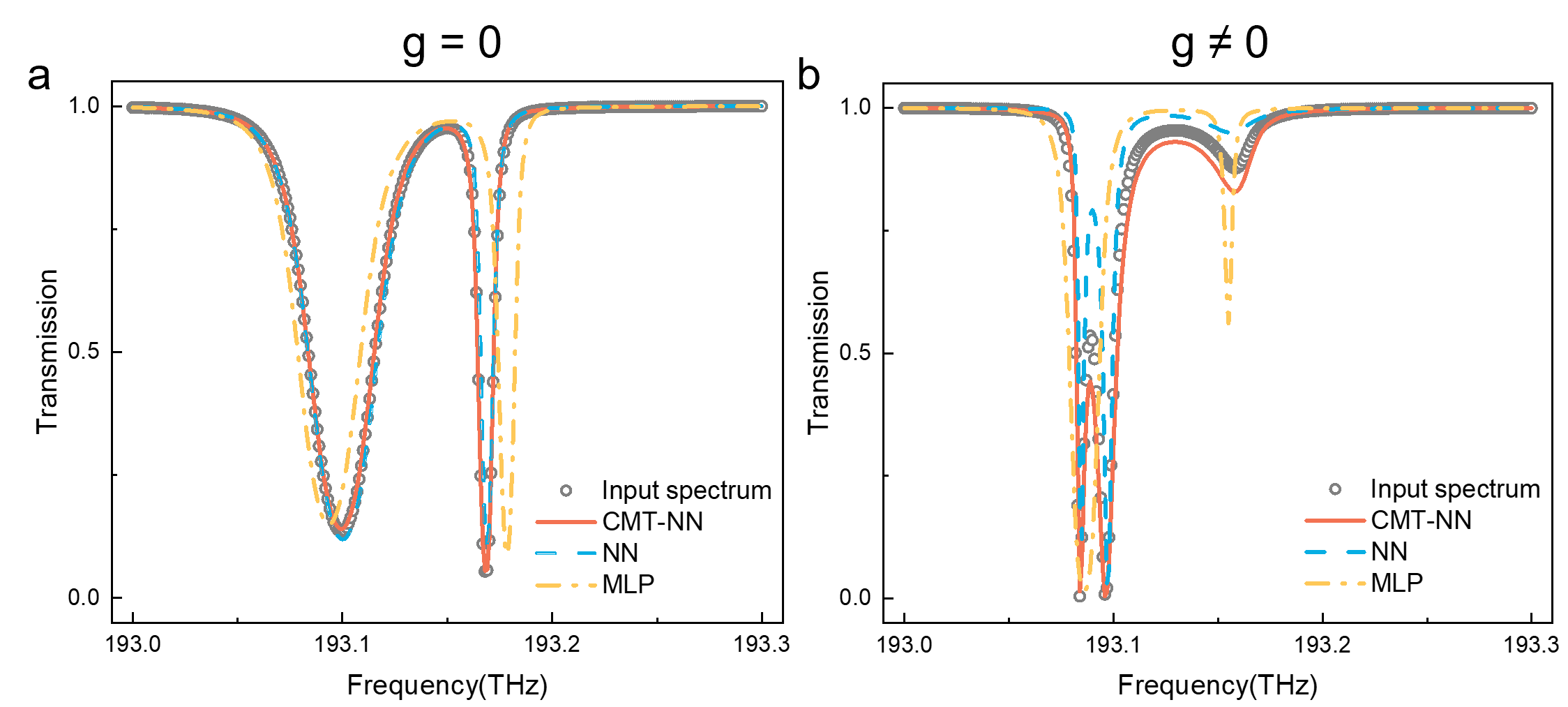


**Figure S9.** **Two typical examples where the reconstructed transmission spectra using the parameters predicted by CMT-NN, NN and MLP for the cases without (a) and with (b) direct coupling.** The circles, red lines, blue lines and yellow lines correspond to the input transmission spectra, reconstructed transmission spectra using the predicted physical parameters of CMT-NN, NN and MLP, respectively.

| Figure S9a | Ground truth | CMT-NN | NN | MLP |
| --- | --- | --- | --- | --- |
| $g$ | 0 | 0 | 2.93464×10^-5^ | 0.00130 |
| Mean absolute error | / | 0 | 2.93464×10^-5^ | 0.00130 |
| Figure S9b | Ground truth | CMT-NN | NN | MLP |
| $g$ | 0.01101 | 0.01126 | 0.00408 | 0 |
| Mean absolute error | / | 2.49671×10^-4^ | 0.00694 | 0.01101 |

**Table S1. The predicted results of parameter *g* by CMT-NN, NN and MLP using the input transmission spectra of Figure S9.**

**Supplementary Note 5 — Performance comparisons between the CMT-NN and the traditional fitting method**

In this section, we compare the performance of CMT-NN, differential evolution quasi-Newton method^5^ (DE+QNM) and quasi-Newton method^6^ (QNM). Because DE+QNM stands out as one of the most robust global optimization algorithms^7^, and QNM is recognized as one of the most efficient and widely used optimization algorithms^8,9^, they are selected as comparison benchmarks for CMT-NN. DE+QNM employs the global search mechanism of differential evolution (DE), using population evolution to extensively explore the solution space and locate potential solutions. Subsequently, QNM performs fine gradient optimization in these regions to achieve efficient convergence.

The comparing results of the three methods are shown in Figures S10 and S11. Here, we assume that the input spectrum is known whether there is a coupling scenario. First, we compare the methods with direct coupling between two microcavities is zero. Figure S10a shows both the CMT-NN and DE+QNM methods successfully reconstruct the spectrum with the same parameter space. However, the QNM method needs to continuously iterate over a range of parameter spaces to achieve the fitting and reconstruction of the input spectrum. As shown in Figure S10, the reconstructed spectra of three methods using their predicted physical parameters by CMT show good consistency with the input transmission spectra, and the required computation time of QNM method is 0.7 s. It should be noted that we have only considered the fitting time of the QNM method for each iteration, excluding the time required for adjusting the fitting range. Therefore, the QNM method not only requires frequent adjustments to the fitting range but also costs significant computation time in practice. As shown in Figure S11, the QNM method needs to adjust the range of fitted parameters five times to match well with the input spectrum when direct coupling is present (i.e., *g* ≠ 0), and the reconstructed spectra at different fitting ranges are shown in Figures S11b-e. The reconstructed spectra of different methods using their predicted physical parameters by CMT show good consistency with the input transmission spectra when ignoring the required fitting range and computation time. However, the DE+QNM method shows the drawbacks in batch spectral fitting (Figs. 4g and 4h of the main text) and solution multiplicities (Table S2) are unavoidable. In addition, the QNM method cannot quantitatively describe the computation time, so we only compare the batch processing capabilities of CMT-NN and DE+QNM methods, as shown in Figs. 4g and 4h of main text.


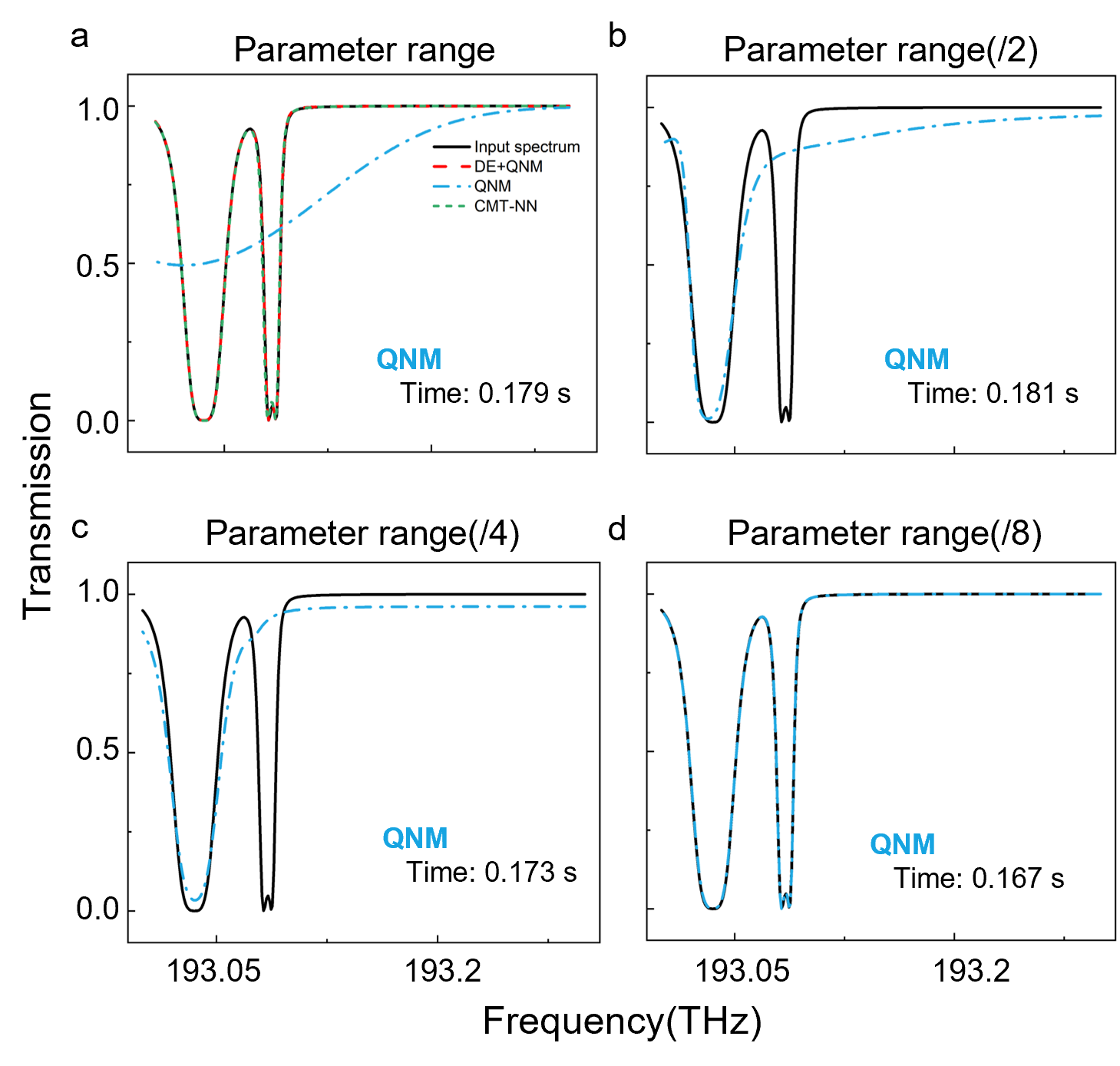


**Figure S10. The comparison of reconstructed transmission spectra among the CMT-NN, DE+QNM, and QNM when *g* = 0. a,** Comparison results of the reconstructed spectra using the predicted parameters by CMT of three methods at full fitting range. **b-d,** The fitting spectra of QNM methods at half range (b), quarter range (c), and eighth range (d) of fitting parameters range. The black, red, blue, and green lines represent the input spectrum, the results obtained by the DE+QNM method, the QNM method, and the CMT-NN method, respectively.


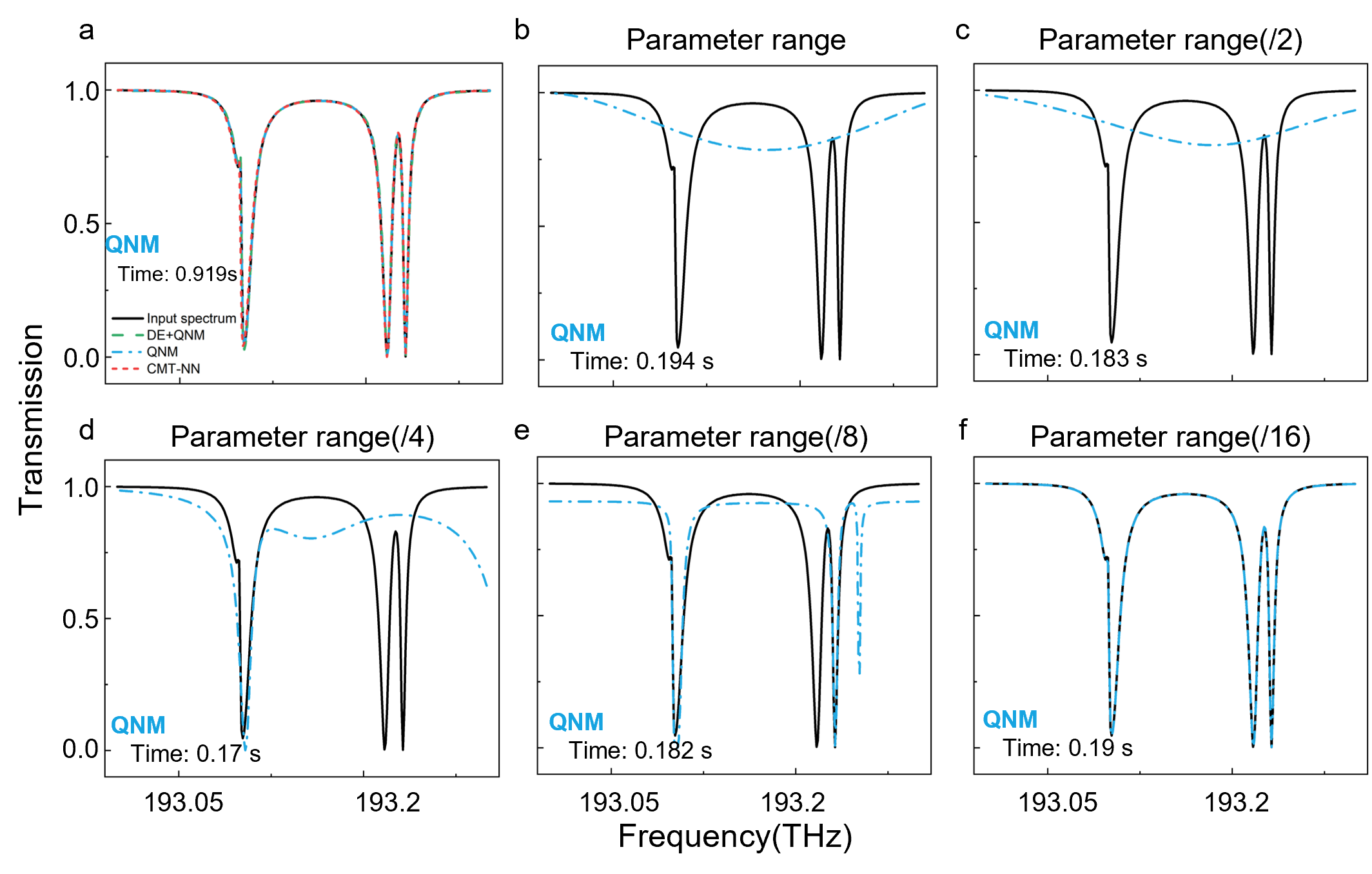


**Figure S11. The comparison of reconstructed transmission spectra among the CMT-NN, DE+QNM, and QNM when *g* ≠ 0. a,** Comparison results for the reconstructed spectra using predicted parameters by CMT of three methods. **b-f,** The fitting spectra by QNM methods at full range (b), half range (c), quarter range (d), eighth range (e) and sixteenth range (f) of fitting parameters range. The black, red, blue, and green lines represent the input spectrum, the results obtained using the DE+QNM method, the QNM method, and the CMT-NN method, respectively.

| Fig.4a | $\omega_{1}$ | $\omega_{2}$ | $\gamma_{c1}$ | $\gamma_{c2}$ | $g$ | $g_{1}$ | $g_{2}$ | $\varphi$ | MSE |
| --- | --- | --- | --- | --- | --- | --- | --- | --- | --- |
| Ground truth | 193.12623 | 193.17665 | 0.01695 | 0.00644 | 0 | 0.00386 | 0.00834 | 4.039 | */* |
| CMT-NN | 193.12550 | 193.17717 | 0.01695 | 0.00645 | 0 | 0.00383 | 0.0083 | 4.0675 | 0.0001 |
| DE+QNM | 193.12697 | 193.17628 | 0.01712 | 0.00634 | 0.00478 | 0.0048 | 0.0073 | 1.6064 | 0.7398 |
| DE+QNM  (g=0) | 193.17673 | 193.12626 | 0.00628 | 0.01695 | 0 | 0.00834 | 0.00382 | 5.4322 | 0.2691 |
| Fig.4b | $\omega_{1}$ | $\omega_{2}$ | $\gamma_{c1}$ | $\gamma_{c2}$ | $g$ | $g_{1}$ | $g_{2}$ | $\varphi$ | MSE |
| Ground truth | 193.14603 | 193.16383 | 0.04145 | 0.0105 | 0.07275 | 0 | 0.00376 | 0.322 | */* |
| CMT-NN | 193.1454 | 193.16333 | 0.04128 | 0.01097 | 0.07285 | 0 | 0.00358 | 0.3643 | 0.0002 |
| DE+QNM | 193.11152 | 193.19833 | 0.02821 | 0.02305 | 0.05857 | 0.0017 | 0.00225 | 0.3898 | 0.0150 |

**Table S2. The parameters’ predicted results of CMT-NN and fitting parameters results of DE+QNM (*g* ≠ 0) and DE+QNM (*g* = 0) for the input spectrum of Figs. 4a and 4b in main text.** The unit of physical parameters *ω*_1_, *ω*_2_, *γ*_c1_, *γ*_c2_, *g*, *g*_1_ and *g*_2_ are THz, and the unit of phase $\varphi$ is rad.

Additionally, when comparing with the traditional methods, we incorporate the pre-training time of CMT-NN framework into the total computation time. It should be noted that the total time required for generating training data and completing model training primarily depends on hardware configurations, dataset size, and training parameter configurations. Detailed descriptions of the dataset composition, training parameters, and hardware configurations are provided in the Methods section of the main text. Under the two coupling scenarios considered in this work (i.e. g = 0 and g ≠ 0), the time costs for data generation and model training are shown in Table S3. Data generation time is substantially shorter than training time. The total duration of CMT-NN is approximately 2.34 hours (g = 0) and 3.04 hours (g ≠ 0), respectively. It is important to emphasize that this training process is a one-time investment. Once trained, the model can be repeatedly applied to rapidly solve similar problems without requiring retraining. For a single transmission spectrum or a small number of transmission spectra, traditional fitting methods do require less time than the CMT-NN when the training time is taken into account. In the case of a large number of transmission spectra, the CMT-NN becomes advantageous in terms of computation time. The balance point is reached at approximately 5,000 and 3,200 transmission spectra under the scenarios of g = 0 and g ≠ 0 using our software and hardware, respectively. Beyond these thresholds, the CMT-NN delivers both shorter computation time and higher prediction accuracy, demonstrating an appealing advantage. This makes the CMT-NN particularly suitable for large-scale data tasks, such as real-time dynamic monitoring and high-throughput device characterization.

|  | Data generation (s) | Model training (s) | Total time (h) |
| --- | --- | --- | --- |
| without direct coupling  (*g* = 0) | 5.11 | 8426.69 | ≈2.34 |
| With direct coupling  (*g* ≠ 0) | 5.45 | 10962.27 | ≈3.04 |

**Table S3. The time cost of data generation and model training for the cases with and without direct coupling.**

**Supplementary Note 6 — Long-term stability of experimental coupled system**

In this section, we test the long-term stability of the experimental coupled system. As shown in Figure S12, rigorous long-term stability characterization of the coupled system by monitoring a specific resonant mode under scanned laser excitation (central wavelength is 1550 nm, scan signal is 50 Hz triangular wave) demonstrates that the contribution of thermal drift to the overall uncertainty is statistically negligible.

**Figure S12. The long-term stability of the experimental coupled system.** The laser is operated with its center wavelength at 1550 nm and is precisely scanned using a 50 Hz triangular wave from an arbitrary function generator.

**Supplementary Note 7 — Investigation of CMT-NN’s robustness under different coupling scenarios**

To evaluate the robustness of CMT-NN, we add Gaussian noise with different standard deviations into the spectra of validation set. Two typical examples are given in Figures S13a-c and S13d-f, the reconstructed spectra of different methods using their predicted physical parameters show good consistency with the input transmission spectra even under noise interference. For the transmission spectra of the validation set, the predicted physical parameters’ mean squared errors of σ = 0.001, 0.005 and 0.01 are 3.52×10^-4^/0.00947, 0.00115/0.01616 and 0.00181/0.016315 in the case of without/with direct coupling, respectively. As can be seen from these results, the proposed CMT-NN demonstrates robustness under certain noisy conditions.

We present the displacement prediction results obtained from microcavities of different geometries and fabrication batches, as shown in Figure S14, where displacement detection is achieved by leveraging the pre-calibrated correspondence between changes in the physical parameters and the displacements. The displacement sensing demonstrates good performance, with corresponding coefficients of determination R² up to 0.9796 (*g* = 0) and 0.9964 (*g* ≠ 0) for the two cases, respectively. These results demonstrate the robust performance of CMT-NN and its strong potential for practical real-world applications.


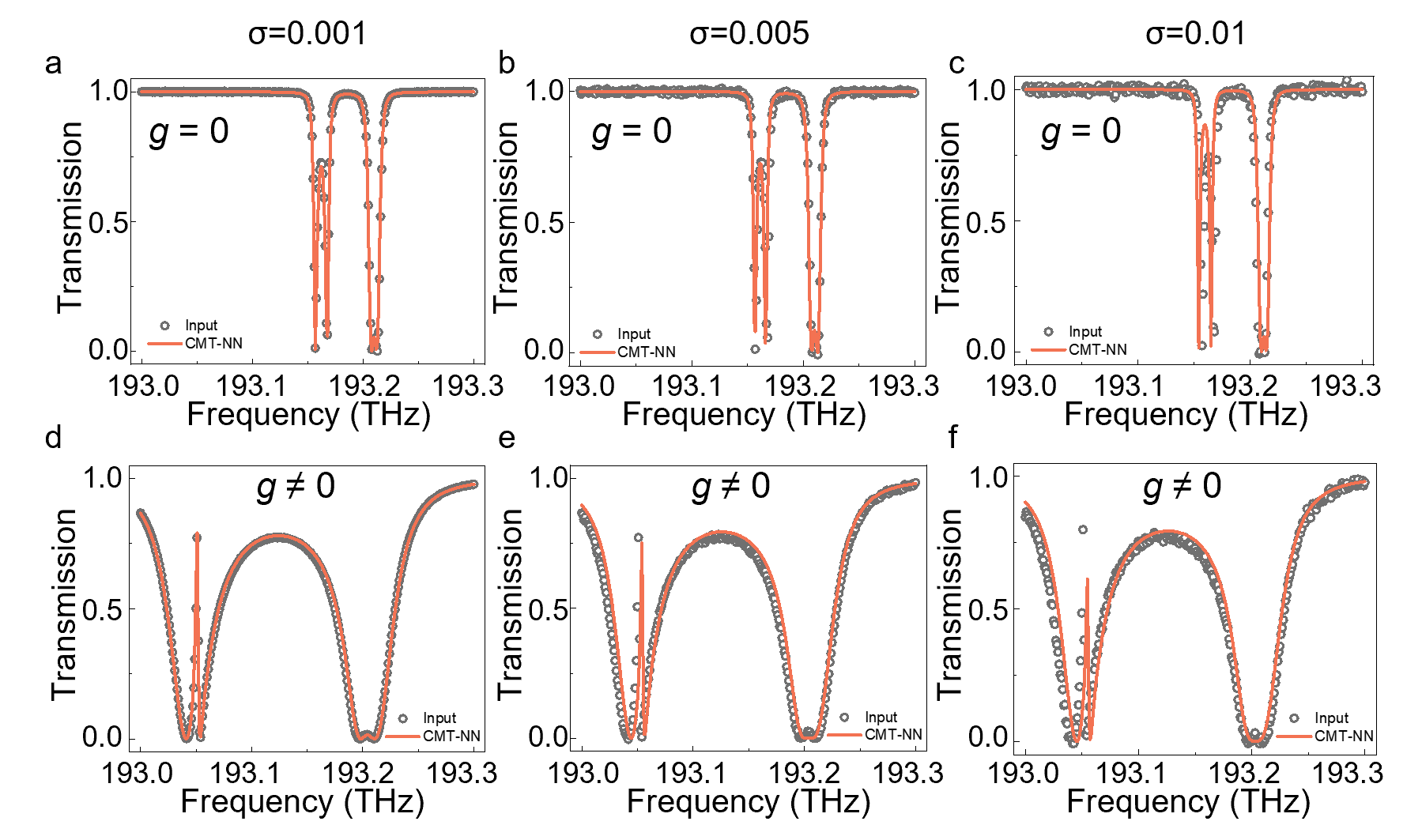


**Figure S13. Robustness of CMT-NN under perturbations with different standard deviations of Gaussian noise. a-c,** The reconstructed spectra using the parameters predicted by CMT-NN for the case without direct coupling (g = 0) under Gaussian noise with standard deviations of σ = 0.001 (a), 0.005 (b) and 0.01 (c). **d-f,** The reconstructed spectra using the parameters predicted by CMT-NN for the case with direct coupling (g ≠ 0) under Gaussian noise standard deviations of σ = 0.001 (d), 0.005 (e) and 0.01 (f).


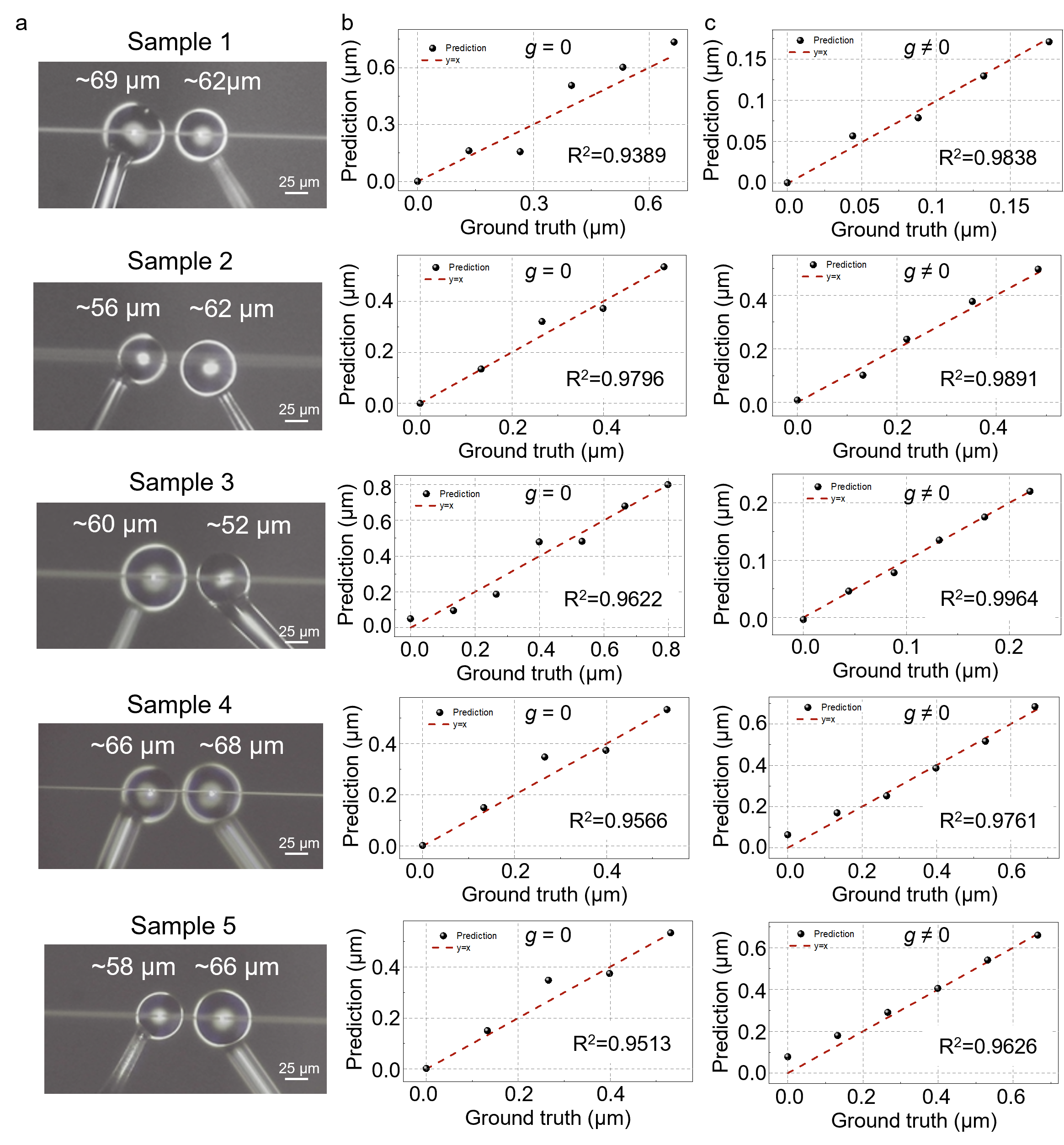


**Figure S14. Dependence of predicted displacement on their ground truths with different geometries.**. **a,** The images of the coupled microspheres resonant systems. **b,c,** Dependence of predicted displacement on their ground truths for the cases that are without (b) and with direct coupling (c). R^2^ is the corresponding coefficient of determination of displacement sensing.

**References**

1. Fan, S. H., Suh, W. & Joannopoulos, J. D. Temporal coupled-mode theory for the Fano resonance in optical resonators. *Journal of the Optical Society of America A* **20**, 569-572 (2003).
2. Yin, S. et al. Deep learning enabled design of terahertz high-Q metamaterials. *Optics & Laser Technology* **181**, 111684 (2025).
3. Lin, M., Chen, Q. & Yan, S. C. Network in network. arXiv preprint arXiv:1312.4400 (2013).
4. Yan, Z. Y. et al. Accelerating reliable multiscale quantum refinement of protein–drug systems enabled by machine learning. *Nature Communications* **15**, 4181 (2024).
5. Zhu, J. P. et al. Parameter estimation for Burr type XII distribution with differential evolution and Quasi-Newton approaches based on progressively type I interval-censored samples. *ICIC Express Letters, Part B: Applications* **8**, 1299-1306 (2017).
6. Dennis, Jr, J. E. & Moré, J. J. Quasi-Newton methods, motivation and theory. *SIAM Review* **19**, 46-89 (1977).
7. Chen, Y. G. et al. Dynamic multi-swarm differential learning particle swarm optimizer. *Swarm and Evolutionary Computation* **39**, 209-221 (2018).
8. Nocedal, J. & Wright, S. J. Numerical optimization. New York, NY: Springer New York (2006).
9. Bottou, L., Curtis, F. E. & Nocedal, J. Optimization methods for large-scale machine learning. *SIAM Review* **60**, 223-311 (2018).
